# Metapresencialidad: concepto fundante de una teoría crítica de la salud digital

**DOI:** 10.18294/sc.2023.4655

**Published:** 2023-10-19

**Authors:** Naomar de Almeida Filho

**Affiliations:** 1 Profesor emérito, Department of Sociology, University of Texas-Austin, EEUU. Presidente, Salud y Fármacos, El Paso, Texas, EEUU. naomaralmeida@gmail.com University of Texas Department of Sociology University of Texas-Austin Texas USA naomaralmeida@gmail.com

**Keywords:** Teoría de la Información, Tecnología Digital, Salud Digital, Telemedicina

## Abstract

En este texto, propongo el concepto de “metapresencialidad” como elemento fundante para una teoría crítica de la salud digital. En primer lugar, presento los conceptos de técnica, tecnología y objeto técnico, centrales en las teorías de Álvaro Vieira Pinto y Milton Santos. En segundo lugar, a partir de la filosofía de la información de Luciano Floridi, cuestiono la pertinencia de la dicotomía real-material-concreto versus digital-virtual-informacional como fundamento ontológico de los conceptos de realidad, lugar y presencia, destacando las nociones de realidad virtual y realidad extendida. En tercer lugar, introduzco una crítica etimológica e histórica de la serie presencia-telepresencia-metapresencia, enfocando la noción emergente de metapresencialidad en forma de protoconcepto y su eventual formalización como fundamento conceptual para una apropiación sociotécnica y una integración tecnosocial de las tecnologías digitales. Finalmente, discuto la salud digital como campo de saberes, técnicas y prácticas y evalúo las ventajas epistemológicas y pragmáticas de la metapresencialidad como concepto en los campos de la informática, la educación y la salud.

## INTRODUCCIÓN

La sociedad que se ha formado a partir de la globalización de los sistemas productivos, desde la segunda mitad del siglo XX, inaugura una nueva fase histórica de la humanidad. Las relaciones de producción en las formaciones sociales contemporáneas han estado marcadas por el uso intensivo y constante de tecnologías, especialmente las tecnologías digitales, en todas las áreas del conocimiento humano y en la acción social^1^. La complejidad del mundo actual determina nuevas formas de intervención en la vida cotidiana, eficientes, ágiles y flexibles, realizadas a través de estrategias sociotécnicas diversificadas, modulares y cambiantes. La emergencia de estas intervenciones en espacios inmateriales y situaciones no presenciales se ha producido a través de sistemas, equipos, procesos y programas de funcionalidad compleja, clasificados como *tecnologías de la información y las comunicaciones* (TIC), con una implementación masiva y generalizada, y un uso cada vez más frecuente en todos los sectores de la economía a escala global. Habilitadas por sistemas de automatización robótica, programados en lenguaje de máquina y controlados por algoritmos (recientemente, por inteligencia artificial), las TIC han llevado a considerar el capitalismo contemporáneo como una “economía digital”^2^.

En el plano de la sociedad, con la difusión mundial de las TIC en todos los ámbitos de la vida social, se observa una creciente proliferación de una variedad de procesos, productos y patrones de uso social de tecnologías digitales para la producción y utilización de datos, información y conocimientos. En este escenario, los dispositivos integrados de comunicación y los sistemas de conexión interpersonal (redes sociales, chats, blogs, etc.) fomentan procesos de integración tecnosocial mediados por interfaces humano-máquina, creando formas de sociabilidad. Como resultado, la organización social humana y los procesos relacionales de la vida cotidiana se vuelven cada vez más dependientes de bases de datos, fuentes de información, redes digitales y dispositivos electrónicos, justificando la etiqueta de “sociedad del conocimiento”^3^ que está de moda.

La masividad de la oferta de objetos técnicos digitales y su uso generalizado se ha considerado como un factor de desempleo estructural, retroceso educativo, exclusión social, alienación cultural y un posible vector de daño a la salud mental^4^. No obstante, los optimistas postulan que, aprovechando las brechas y oportunidades, los procesos políticos de racionalidad democrática y la educación interactiva, adecuados y llevados a cabo de manera competente, podrían reducir los riesgos y compensar los efectos perjudiciales del tecnocentrismo^5^. De esta manera, se cultiva la esperanza de que las TIC, con sus múltiples potencialidades, puedan contribuir a la formación ciudadana completa de un nuevo sujeto epistémico, estimulado a aprender a lo largo de la vida, en relación solidaria con comunidades humanas convertidas en virtuales y con un entorno sostenible^6^.

En todo el mundo, la apropiación sociotécnica de conocimientos y experiencias de intervención basadas en TIC ha propiciado la implementación de ecosistemas de salud innovadores que, más que incrementos, complementos o accesorios a formas, modelos, estrategias y métodos existentes, potencialmente representan una revolución en los modos de atención médica^7^. Con la pandemia de covid-19, en los campos de la educación y la salud, ha aumentado considerablemente el interés por las tecnologías digitales que producen percepciones inmersivas, sustituyendo la presencia material por formas sensoriales de presencia remota. Desde entonces, las *tecnologías digitales en salud* (TDS) se han difundido en situaciones y contextos que movilizan grandes contingentes de operadores técnicos y un enorme volumen de información, junto con la proliferación de objetos técnicos relevantes. El recorte de este conjunto de objetos técnicos, de técnicas, de innovaciones tecnológicas y sus operadores, organizados y activos en espacios, redes institucionales y comunidades de práctica, se ha convertido en un nuevo campo social que puede denominarse *salud digital*.

Para abordar este conjunto de cuestiones de manera rigurosa y consistente, propongo explorar fundamentos y sistematizar conceptos necesarios, útiles y viables para la construcción de una teoría crítica de la salud digital, desde una perspectiva de desobediencia epistémica^8^. En este artículo, destaco uno de estos conceptos: la metapresencialidad, como punto focal de reflexión capaz de configurar estrategias y calificar oportunidades de aplicación de las TDS. En cierta medida, aunque de manera preliminar y limitada en alcance, este texto representa un esfuerzo por ampliar, organizar y detallar una breve comunicación personal incluida en el libro *O futuro começa agora: Da pandemia à utopia* de Boaventura de Sousa Santos^9^.

En este proceso de construcción teórica compartida, en primer lugar, propongo presentar las nociones de tecnología, técnica y objeto técnico, basándome en la contribución teórica de Álvaro Vieira Pinto^10^^,^^11^ y Milton Santos^12^^,^^13^. En segundo lugar, en diálogo con la filosofía de la información, propuesta por Luciano Floridi^14^^,^^15^^,^^16^^,^^17^, a partir de una teoría del modo de producción de saberes tecnocientíficos^18^^,^^19^^,^^20^^,^^21^, cuestiono la pertinencia de la dicotomía real-material-concreto *versus* digital-virtual-informacional como base epistemológica para definir los conceptos de realidad, lugar, presencia y presencialidad, destacando las nociones de realidad virtual y realidad extendida. En tercer lugar, introduzco una perspectiva de crítica etimológica e histórica de la serie semántica presencia-telepresencia-metapresencia, focalizando la noción emergente de metapresencialidad en forma de protoconcepto y su eventual formalización como fundamento conceptual para una apropiación sociotécnica e integración tecnosocial de las TDS. Finalmente, en comparación con las propuestas del metaverso (en el campo de la computación) y las nociones convencionales de educación a distancia (en el campo de la educación) y telemedicina (en el campo de la salud), evalúo el potencial de ventajas epistemológicas, heurísticas y operativas del concepto de metapresencialidad para la constitución de la salud digital como campo de conocimientos, técnicas y prácticas.

## TECNOLOGÍA, TÉCNICA, TECNOCENTRISMO

Álvaro Vieira Pinto (1909-1987) fue un polímata (médico, matemático, físico, demógrafo, traductor, filósofo, pensador social y educador), líder de una generación de intelectuales representativos del pensamiento nacional-desarrollista de izquierda a fines del siglo XX. En su vasta obra, Vieira Pinto^10^^,^^11^ propone un análisis filosófico, histórico y político sobre las relaciones entre trabajo y producción, naturaleza y técnica, ciencia y cultura vinculadas al proceso de desarrollo dependiente. Sus reflexiones críticas sobre los fenómenos relacionados con la incorporación y apropiación social de técnicas y tecnologías han sido rescatadas y estudiadas, principalmente después de la publicación póstuma de *O conceito de tecnologia*^11^. En esta obra, un ambicioso y complejo tratado sobre la era tecnológica y sus desarrollos, comienza por deconstruir la expresión “era tecnológica”, ampliamente difundida en ese momento, utilizando un argumento directo y contundente para criticarla y refutarla: precisamente porque son humanos, los seres humanos siempre han vivido en eras tecnológicas. Al producir tecnologías cada vez más complejas y sofisticadas, el ser humano se vuelve más dependiente de ellas, en una relación dialéctica y tendencialmente conflictiva. En la actualidad, la tecnología desempeña un papel indispensable en el funcionamiento social y en las relaciones laborales, facilitando la reducción de los problemas del progreso tecnológico a aspectos exclusivamente “técnicos”^11^.

En el imaginario contemporáneo, como una construcción ideológica, el término “tecnología” constituye una metonimia (más precisamente una sinécdoque), utilizada para referirse a cosas y temas muy diversos entre sí: objetos técnicos materiales (herramientas y equipos electrónicos), operados por técnicas (programas y protocolos fijos o autoprogramables) y, como condición para la viabilidad de estos objetos y procesos, tecnologías digitales de información y conectividad. Para superar esta ideología tecnológica, Vieira Pinto^11^ identifica la necesidad de una mayor precisión en la conceptualización de lo que es la “tecnología”, distinguiéndola de los conceptos de técnica, de instrumento y de producto (que luego se conceptualizaron como “objeto técnico”). Para él, el término “técnica” se refiere a la forma en que se realizan los actos productivos del ser humano, materializándose en instrumentos, máquinas y artefactos que transforman la naturaleza, humanizándola a través de la cultura.

Por su parte, el término “tecnología” se despliega en dos conceptos y tres usos de sentido común, con cierto grado de superposición desde el punto de vista semántico. Primero, en una referencia teórica etimológicamente precisa, el concepto de tecnología significa la ciencia de la técnica (*technê* + *logos*) o el conocimiento sobre la técnica. Segundo, en el discurso social común, en el cual Vieira Pinto^11^ destaca una visión algo ingenua sobre la “tecnología”, la noción de tecnología a menudo se reduce a la técnica o conjuntos de técnicas, equiparando proceso y discurso. Tercero, como derivación de esta connotación lega, la concepción antropológica de tecnología se refiere a todo el conocimiento sistemático producido y acumulado históricamente por el hombre a lo largo de su existencia, y comprende el conjunto de técnicas desarrolladas y apropiadas en un período histórico determinado^10^. Cuarto, la concepción dominante sobre la naturaleza de la tecnología, representativa de un pensamiento acrítico y anacrónico, se refiere a la ideología de la técnica^11^. La concepción de la técnica como ideología permite una comprensión crítica del tecnocentrismo, definido por Seymour Papert^22^ como la sobrevaloración de la tecnología, colocándola en el centro de la actividad humana y dándole la importancia de “principal solucionador” de los problemas de la humanidad. Esta última acepción de la técnica tiene que ver con el imaginario social del mundo contemporáneo, capaz de convertir la tecnología en mitología, tal como indicó Vieira Pinto^11^. El tecnocentrismo se presenta al sujeto humano alienado, al no reconocer que:

…la máquina no es más que su obra, producto de sus fines interiores, [...] y cree que, por el contrario, debe dejarse poseer por la tecnología, porque solo así podrá adquirir un nombre y una esencia humanos, la de técnico”. ^11^

Para deconstruir esta trampa ideológica del tecnocentrismo alienante, en un tono apasionado y militante, Vieira Pinto^11^ nos alienta a:

…romper el círculo infernal de una falsa totalidad en la que los dominadores quieren encerrarnos, bajo el pretexto de que todos participamos en el mismo mundo, unificado por la ciencia y la técnica, que han llegado ahora a un grado de progreso tal que nadie puede rechazarlas, pero tampoco tiene derecho a dar rienda suelta a la creación por su cuenta.

Con el fin de acercarnos críticamente a los conceptos, prácticas, estrategias y dispositivos de las tecnologías digitales, también podemos recurrir a la teoría de la técnica en el ámbito social del geógrafo, epistemólogo y pensador crítico Milton Santos [1926-2001]. En su esfuerzo por recrear la epistemología de las ciencias humanas y sociales en su conjunto, Milton Santos^12^^,^^13^ propone un enfoque que considera el espacio como un conjunto inseparable o una totalidad de sistemas de objetos y sistemas de acciones. El espacio es una mezcla, un híbrido, un complejo, un entorno geográfico compuesto por diferentes formas y contenidos concretizados en múltiples totalidades. Lo que siempre ha existido a partir de estas totalidades es un entorno geográfico que se transforma históricamente, el cual durante dos o tres siglos fue llamado “entorno técnico o maquínico” y que hoy en día podemos designar como “entorno técnico-científico-informacional”.

Para Milton Santos^13^, la “forma principal de relación entre el ser humano y la naturaleza o, mejor dicho, entre el ser humano y su entorno, se da a través de la técnica”. Como base para esta afirmación, las técnicas se entienden como un conjunto de medios instrumentales y sociales a través de los cuales el ser humano lleva a cabo su vida, produciendo y al mismo tiempo creando espacio. En la teoría de Milton, la relación entre el espacio y el fenómeno técnico abarca todas las manifestaciones de la técnica, incluyendo las técnicas de la propia acción, especialmente aquellas que producen objetos técnicos. Se trata de abordar el fenómeno técnico como una totalidad compleja, evitando que nos dejemos deslumbrar por técnicas definidas de manera abstracta. No se trata solo de considerar “las llamadas técnicas de producción o, como otros prefieren, las técnicas industriales, es decir, la técnica específica vista como un medio para lograr un resultado específico”^13^.

Milton Santos^13^ enfatiza la importancia de distinguir entre técnicas particulares -cuando se examinan en su singularidad- y la técnica como fenómeno total. En consecuencia, no se puede concebir una separación rígida entre “un entorno geográfico, por un lado, y un entorno técnico, por el otro”^13^. Así, en el “entorno técnico-científico-informacional”, las técnicas deben ser vistas no solo en su aspecto material, sino también en sus aspectos inmateriales, a través de su propia historia como sistemas que marcan épocas. Santos continúa:

Para discutir el presente y las condiciones actuales de realización y transformación del espacio, supongo desde el primer momento el conocimiento de lo que constituye el sistema técnico actual y cómo, basándose en las condiciones de la técnica actual, una técnica informacional, se establecieron las condiciones materiales y políticas que autorizaron la producción de una inteligencia planetaria.

En el contexto contemporáneo, las tecnologías de la información digital son fundamentales para recrear y demarcar nuevos paisajes geográficos y geopolíticos. En este sentido, comenta que “la información hoy desempeña un papel análogo al desempeñado en el pasado por la energía” al convertirse en la herramienta para unir diferentes partes de un territorio abstracto que, gracias a la cobertura informativa, se ha vuelto menos local y más global, permitiendo la “presencia de cuerpos ausentes”^13^. Aprovechando los avances en movilidad y conectividad proporcionados por la tecnología de la información, que define el mundo contemporáneo, las clases dominantes, paradójicamente, participan cada vez menos en el mundo local de los territorios y, por lo tanto, “ven poco de la ciudad y del mundo”^12^.

En este contexto complejo y cambiante, la técnica desempeña un papel central, y la tecnología es uno de los principales sistemas de acciones presentes en los territorios del mundo globalizado por la hegemonía cultural del occidente capitalista. En esta etapa actual del capitalismo, donde las TIC actúan como mecanismos organizadores de la fabricación, distribución y comercialización de bienes, productos y servicios, el adjetivo “digital” (del latín *dígitus*, que significa dedo, ya que contar con los dedos fue el principal método primitivo utilizado para computar cosas) se ha utilizado en el sentido de número, para designar sistemas y procesos producidos a partir de la codificación de señales, datos e información, así como cualquier efecto que estos produzcan^11^. Por lo tanto, el término “tecnología digital” se refiere a técnicas (procedimientos, protocolos, directrices) y objetos técnicos (equipos, dispositivos) cuya funcionalidad y operación efectiva dependen de programas y lenguajes (sistemas operativos, lenguaje de programación, algoritmos) habilitados por sistemas lógicos o secuencias de comandos formulados en códigos binarios.

Con el desarrollo de formas y dispositivos de digitalización, compresión e integración de señales, imágenes y sonidos, el adjetivo “virtual” se ha utilizado para nombrar tecnologías de creación de entornos artificiales o simulados a través de medios inmersivos. El rápido avance de estas tecnologías, especialmente en los sectores de entretenimiento (juegos) y formación (simuladores), ha ampliado la capacidad de procesamiento, compresión, transmisión e integración de señales, y ha generado dispositivos cada vez más eficientes desde el punto de vista sensorial, difundiendo soluciones tecnológicas que producen efectos de simulación y modelado de entornos y objetos, relacionados con simulacros sensoriales conocidos como “realidad virtual”. Entre otras funciones y posibilidades, estos objetos, situaciones y estados permiten redefinir la noción misma de virtualidad como propiedad de espacios, objetos, sistemas y procesos modelados mediante códigos y sintaxis digitales. A partir de una evaluación de estos contextos tecnológicos, propongo explorar la hipótesis de no pertinencia o invalidez de la dicotomía casi intuitiva, omnipresente en el sentido común, que contrasta los términos real-material *versus* digital-virtual, contrapuestos en términos absolutos y excluyentes. Desde un punto de vista filosófico, se trata de una cuestión ontológica fundamental que analizaré en la próxima sección.

## REALISMOS, REALIDADES, VIRTUALIDADES

Para fundamentar este análisis, inicialmente busqué realizar una revisión crítica de la obra del filósofo italiano Luciano Floridi^14^^,^^15^^,^^16^^,^^17^, quien formuló una filosofía de la información basada en lo que se podría denominar “realismo ontológico de la información”. Esta propuesta abre y organiza todo un campo de investigación sobre categorías y conceptos que, en el mundo contemporáneo, se centran en los condicionantes, procesos e impactos de la cibernética y las tecnologías de la computación, con un enfoque en las ciencias de datos y la ciencia de la información. En sus propias palabras^15^, se trata de un:

…campo filosófico que comprende la investigación crítica de la naturaleza conceptual y los principios de la información, incluyendo sus dinámicas, usos y ciencias, y la elaboración y aplicación de metodologías teóricas-informacionales y computacionales para abordar problemas filosóficos.

El repertorio de problemas investigativos enumerados por Floridi^14^^,^^15^ abarca cinco grupos de temas que serían propios de una filosofía realista de la información: información, semántica, cognición, ética y ontología.

Al abordar la cuestión ontológica fundamental de ¿qué es la información?, Floridi^15^ señala la imposibilidad y, en última instancia, la falta de relevancia de una teoría unificada de la información. En este proceso, identifica tres concepciones de la información con un claro sesgo ontológico: a) información como realidad, como en la cibernética; b) información sobre la realidad, que sería el caso de la información semántica; c) realidad como información, de la cual el genoma sería un ejemplo. Como eje central de su proyecto filosófico, Floridi prioriza las concepciones de la información como realidad o sobre la realidad, que se desarrollan en seis enfoques:

- teoría matemática de datos/señales: define la información en términos de formalización numérica;
- enfoque probabilístico: define la información en términos del espacio estocástico;
- enfoque topológico: define la información en términos del espacio modal;
- modelado sistémico: define la información en términos de procesos y flujos;
- enfoque inferencial: define la información en función del espacio de inferencias;
- enfoque semántico: se revela como el objetivo principal de su investigación filosófica, habilitada como objeto analítico-sintético, al definir la información en términos del espacio de datos.

Para formular una teoría semántica de la información, Floridi^14^ propone delimitar los conceptos de la ontología de la información a partir de sus atributos respectivos: la información debe ser cuantificable, plausible, acumulativa, almacenable y transmisible. La naturaleza semántica de la información no es exclusiva ni necesariamente lingüística, y se observa la independencia de la información semántica del medio físico, el formato y el lenguaje. En síntesis, los datos verificados o validados, estructurados lo suficiente como para ser codificados en bases numéricas y, lo más importante de todo, significativos, son los que permiten concretar la información semántica^14^. La materia prima de la información es el dato, transformado según una sintaxis, siguiendo las reglas de un sistema, código o lenguaje disponibles para el sujeto operador. Los datos, a su vez, emergen de “redes de observables”^15^, anclados en marcos ontológicos, cuya verificación o validación implica compromisos epistémicos que permiten construir redes de información y matrices conceptuales.

En el ámbito de la cognición humana, los datos aparecen como una condición para la producción de información, ya que al configurar conocimientos capaces de guiar técnicas y respaldar prácticas, la información se convierte en una condición constitutiva del conocimiento y su validez pragmática. Surge aquí la cuestión de cómo los datos significativos convertidos en información alcanzan el valor de la verdad, lo que abre espacio para una teoría de la verdad basada en la semántica de la información como materia prima del conocimiento. En esta dimensión, Floridi^17^ propone un “mapa de información semántica” como una sucesión transformadora que define lo que se llama “inteligencia”. En consecuencia, surge la cuestión de si la cognición puede entenderse en términos de procesamiento de información o si se necesita una transformación interpretativa para pasar de la información al conocimiento.

En el plano ético-valorativo, para Floridi^15^, las tecnologías de la información tienen la capacidad de afectar a los sistemas sociales y las formas de vida cotidiana. A la luz de la filosofía de la información, se requiere una ética de la computación para abordar las demandas normativas que surgen de la dinámica de la información, que a menudo produce efectos no deseados y, a veces, perjudiciales. Esto puede ocurrir de dos maneras: por un lado, identificando problemas emergentes con el fin de prevenirlos o al menos generar conciencia entre profesionales, políticos y la opinión pública; por otro lado, aplicando medidas correctivas para reparar los efectos negativos de la dinámica de la información y los problemas sociales producidos por las nuevas tecnologías. En este sentido, Floridi^15^ menciona microéticas construidas en torno a algún valor moral de la información: primero, considerándola como un recurso útil; segundo, considerándola como un producto utilizado para generar más información; tercero, en una perspectiva histórica del contexto de la información que se entrelaza con los contextos culturales, sociales y políticos.

Finalmente, en el plano ontológico, especialmente en lo que respecta a la relación entre datos y naturaleza y entre información y realidad (mundo natural), la semántica de la información de Floridi^14^^,^^15^ pretende evaluar la calidad de esta relación. Para ello, explora la posibilidad de que la naturaleza misma del mundo natural haya configurado ecosistemas de información como una infosfera (infoesfera), un espacio de realidades y temporalidades simultáneamente materiales y virtuales, naturales e informacionales. De ahí que, de muchas maneras, sea justificable hablar de tiempo real en referencia a la simultaneidad o sincronía de la interacción digital. Floridi^15^^,^^16^ desarrolla la idea de niveles de abstracción para proponer la modelización como un posible vínculo entre lo real y los procesos de semantización, que son, en última instancia, procesos de construcción de la realidad. Como vimos anteriormente, para convertirse en información, los datos necesitan adquirir significado, por lo tanto, la modelización de la información produce efectos semánticos en realidades construidas^17^. De esta manera, según lo analizado por González^23^, la tesis del realismo informacional de Floridi comprende el proceso de modelización como una interfaz dialéctica entre los datos (que respaldan los procesos de semantización de lo real) y la información (que vincula la semantización a procesos pragmáticos capaces de generar conocimiento).

Con la intención declarada de superar epistemologías de la representación y la interpretación heredadas del racionalismo cartesiano y sus derivaciones, Floridi^24^ adopta una perspectiva constructivista muy propia y peculiar. Al postular que la vida humana está ligada a eventos relevantes en el mundo del lenguaje, supone que este vínculo autoriza a lo simbólico (el núcleo del lenguaje humano) a separar la realidad de lo real. Capurro^25^ cuestiona la tesis de Floridi de que, constituido como operador semántico, el ser humano sería capaz de promover una división entre cosa y símbolo a través de la mediación lingüística. Esta postulación ya había sido evaluada (y criticada) como metafísica y neoplatónica. En cualquier forma de platonismo, ya sea clásico o contemporáneo, lo real se define como lo que estaba establecido como un límite, y las realidades se construyen para tratar de dar cuenta de lo real que, de manera siempre restringida y parcial, se presenta para la semantización. En contraste, el enfoque naturalista aristotélico, heredado por el empirismo dominante en las ciencias naturales, define lo real como lo que es y como lo que está, negando cualquier referencia válida al mundo ideal de las formas puras o paradigmas. De diversas formas, el realismo estructural de la información propuesto por Floridi sostiene que el conocimiento se construye a partir de la información, asumiendo que la validez de la información depende de procesos de modelización basados en datos que, a su vez, se originan en observables reales. Esta cuestión reposiciona la problemática de los seres y las conexiones en el universo físico; se trata de verificar si los procesos considerados como naturales, como la causalidad o la presencia temporal, de hecho, comprenden dinámicas de información en una realidad empírica. Para Floridi^14^, esta problemática se desglosa en preguntas cruciales, siendo la principal de ellas la diferencia ontológica entre realidad material y realidades virtuales.

Dada la complejidad de estos temas y con el objetivo de al menos organizar formas de comprensión de las realidades según Floridi, que puedan orientar técnicas y fundamentar prácticas basadas en una virtualidad establecida a través de medios digitales, podemos considerar el siguiente glosario común^26^:

- Realidad restringida: se refiere a un entorno en el que el proceso de atención se limita a una relación terapéutica directa y sincrónica, con la presencia física de los sujetos en todo momento del proceso de atención, sin mediación tecnológica, información o conectividad. Ejemplos de este concepto de realidad restringida incluyen el entorno social tecnocientífico y micropolítico de laboratorios, observatorios y campos de investigación, así como el entorno asistencial de hospitales, clínicas especializadas, servicios ambulatorios y lugares de práctica, que requieren la presencia física de los actores en las etapas cruciales del proceso y no hacen uso de dispositivos digitales.
- Realidad proyectada: se refiere a la reproducción de entornos de atención o de enseñanza-aprendizaje en ubicaciones remotas, donde la relación pedagógica o de demostración puede ocurrir de manera residual o asincrónica, posibilitada por el uso de dispositivos de TIC. Esto implica la mediación tecnológica para la edición, el montaje, el almacenamiento y la transmisión de contenidos preprogramados. Ejemplos de esta modalidad incluyen teleclases o debates grabados en video, editados e ilustrados, que proyectan en el tiempo y el espacio una imagen, un proceso, una situación clínica o un contexto pedagógico.
- Realidad aumentada o extendida: se refiere a la extensión del entorno físico en el que se llevan a cabo relaciones de producción, comunicación, educación, atención y otras formas de sociabilidad relacional, habilitadas por el uso de dispositivos digitales de conectividad^26^. En el campo de la salud, se define como una extensión del entorno real y concreto de atención, donde la relación terapéutica se lleva a cabo de manera directa y sincrónica, y puede ocurrir mediante una presencia virtual (o telepresencia), posible gracias al uso de dispositivos digitales de telecomunicación para la transmisión de contexto, imágenes y sonido. Un ejemplo simplificado de esta modalidad de entorno sería la transmisión en tiempo real de un procedimiento quirúrgico, una conferencia o un espectáculo en pantallas y sistemas de sonido, en salas contiguas al lugar del evento o simultáneamente en lugares remotos, lo que puede ser aún más efectivo en términos educativos si se incluyen dispositivos de participación o interacción.
- Realidad virtual: se trata de un entorno simulado que está desvinculado de una matriz material concreta y es completamente digital. Sus referencias microecológicas se convierten en señales a través de códigos digitales que, al ser decodificados y convertidos nuevamente en estímulos sensoriales, permiten una relación experiencial o motivacional de inmersión^26^. Ejemplos de dispositivos de RV pueden incluir juegos de rol, videojuegos y simulaciones programadas entre avatares. Estos pueden convertirse en simulacros de microentornos anatómicos, fisiológicos, celulares y moleculares especialmente efectivos para la formación técnica y profesional en el campo de la salud.

Este es un tema relacionado con la creación de realidades inmateriales^27^. Tanto la realidad aumentada como la realidad virtual implican una territorialidad deslocalizada, lo que permite una relación financiera, pedagógica o terapéutica en contextos de inmersión sensorial o motivacional, programada por dispositivos y sistemas de producción, condensación e integración de contexto, imagen, sonido y datos^28^. Como resultado (o alternativamente), los procesos de producción de información semántica (en la terminología de Floridi) hacen posible la producción de conocimiento y, en ciclos paralelos, la producción de tecnologías, especialmente tecnologías digitales capaces de proporcionar la virtualidad y sus realidades.

En la práctica, con la llegada de las tecnologías digitales de inmersión sensorial a través de la integración de información audiovisual, modelos alternativos de realidad, una vez más, muestran la falta de pertinencia o futilidad de la distinción entre lo real y lo virtual, señalando su superación a través de formas complementarias, híbridas o transgresoras de esta perspectiva originalmente conjuntista-identitaria^29^. En esta dirección, Mingers y Standing^30^ señalan que las teorías de la información actuales aún tienen un largo camino por recorrer y enumeran una serie de preguntas fundamentales que deben responderse:

¿Cuál es el estatus ontológico de la información?: exactamente qué es ¿una cosa, un concepto, una relación, un significado? ¿Es objetivo, y existe independientemente de observadores o receptores, o es subjetivo, creado en la mente de los observadores al recibir un mensaje? […] ¿Puede haber “información ambiental”, es decir, señales dentro del entorno que llevan información sin la participación de seres humanos? ¿La información tiene que ser verdadera para ser información (una versión verídica), como sostienen Dretske y Floridi? […] ¿Una teoría de la información distingue claramente entre los conceptos relacionados de dato, información, conocimiento y significado? [Traducción libre de: *What is the ontological status of information - what exactly is it - a thing, a concept, a relation, a meaning? Is it objective, existing independently of observers or receivers, or is it subjective, created in the mind of observers on receipt of a message?* […] *Can there be “environmental information”, that is signs within the environment that carry information without the involvement of humans? Does information have to be true to be information (a veridical version) as Dretske and Floridi maintain?* […] *Does an information theory distinguish clearly between the related concepts of data, information, knowledge and meaning?*]

La visión contextualizada identifica un gran potencial en el realismo ontológico informacional para abordar desafíos epistemológicos y situaciones prácticas en el actual escenario tecnocientífico. Con el objetivo de ser aceptado en el *establishment* eurocéntrico, la aproximación del constructivismo informacional de Floridi puede que oculte sus raíces lejanas en una teoría del modo de producción del conocimiento, como sutilmente señala Mingers^31^. El realismo dialéctico-crítico comprende una teoría del conocimiento y la práctica científica que se deriva del materialismo dialéctico y el pragmatismo metodológico (en la línea de Peirce-James-Dewey-Rorty), tal como fue formulado de manera sistemática por el filósofo y lógico indo-británico Ram Roy Bhaskar (1944-2014) a finales de la década de 1970^18^. Otros autores importantes en este movimiento global a favor de un realismo crítico son el lógico estadounidense Donald Mertz^32^, el filósofo australiano Alan Chalmers^33^ y el epistemólogo argentino Juan Samaja^20^^,^^21^.

A pesar de que Floridi no reconoce ningún vínculo teórico con el realismo crítico, en ninguna de sus versiones, su obra implícitamente contiene una teoría de los modos de producción de información-conocimiento con notables similitudes con la epistemología crítica de Juan Samaja (1941-2007). Para Samaja^20^^,^^21^, los atributos de los eventos o fenómenos no son realmente los elementos cruciales para construir el objeto-modelo, el marco teórico que permite la producción de conocimiento científico, sino la praxis metodológica y analítica de las ciencias, guiada por los límites y barreras (o condicionantes) de la realidad concreta. Desde la perspectiva de Samaja^21^, por analogía con la teoría del modo de producción económica desarrollada por el materialismo histórico, el modo de producción tecnocientífico comprende un proceso productivo de conceptos, modelos, bienes y valores definido por propiedades específicas que lo diferencian de la producción de bienes y productos en general.

Desde la perspectiva tanto de Bhaskar^19^ como de Samaja^20^, la práctica científica implica una dialéctica fundamental entre el conocimiento sistemático consolidado como teoría, a través de conceptos organizados y articulados en matrices o modelos explicativos, y los problemas generados por la permanente referencia al campo práctico-empírico, es decir, en una estrecha e inevitable interacción con lo concreto y real. La construcción de teorías consistentes, basadas en conceptos filosóficamente sólidos y contextualmente relevantes, es fundamental para impulsar la producción de conocimiento en diversas áreas de estudio y derivar tecnologías capaces de promover avances en diferentes esferas de aplicación. Según Samaja^21^^,^^34^, el desarrollo del conocimiento científico y tecnológico se produce a través de una cadena productiva que implica la transformación de datos en conceptos, compuesta por etapas de transformación del objeto científico y sus productos intermedios, así como de sus resultados en forma de objeto tecnológico. Esta etapa crucial está mediada por indicadores y metodologías específicas que varían según el tipo de datos utilizados. De esta manera, los conceptos actúan como herramientas heurísticas para comprender los fenómenos estudiados, permitiendo la generalización de la explicación y la aplicación de los hallazgos en diferentes contextos^21^.

Siguiendo el marco de referencia de Bhaskar-Samaja, me alejo de las numerosas concepciones de “información” que prevalecen en las epistemologías del norte global, en particular de la noción empírica de información como contenido transportado por señales o signos en la teoría matemática de la información^35^. En línea con Høstaker^27^, rechazo la idea de la materialidad de la información en el mundo concreto, que es el fundamento de las teorías inspiradas en el giro semántico del neopragmatismo que han dominado el campo de las llamadas “ciencias de la información”^36^^,^^37^. Por otro lado, veo como prometedoras las aproximaciones basadas en el realismo crítico que permiten una comprensión más profunda de los fenómenos sociohistóricos y que posibilitan la integración de información de diferentes áreas, la identificación de patrones y tendencias, y el establecimiento de conexiones entre conceptos y teorías como un modo de producción peculiar que se estructura en ciclos de transformación de datos a información y conocimiento, y luego de saber a técnica y praxis^34^.

La transición de datos a información está determinada por procesos de transformación analítica, que implican una compleja organización, indexación, clasificación, condensación e interpretación de datos. El objetivo de estos procesos es identificar similitudes en dimensiones, atributos, predicados y propiedades entre los casos, con el fin de convertirlos en “información”. Para que los datos se vuelvan relevantes y significativos, es necesario compararlos, buscando patrones y relaciones que generen significado. A través de este proceso, los datos se transforman en “información semántica”, que representa un nivel más alto en el proceso de producción de conocimiento. Como menciona Mingers^31^, esto puede expresarse en la fórmula: información = datos + significado, en un modelo de ciclos de producción de conocimiento. Uno de los primeros modelos de este tipo fue el modelo *data-information-knowledge-wisdom* (DIKW) desarrollado por Ackoff^38^. Desde esta perspectiva, la información se produce a partir de datos que se procesan de manera adecuada y consistente, con el objetivo de resolver problemas, responder preguntas o probar hipótesis^34^.

Para que la información se convierta en conocimiento, las informaciones derivadas de los datos pueden interpretarse, relacionarse con teorías existentes, confrontarse con otros datos y contextualizarse en un marco conceptual más amplio. La transición de la información al conocimiento está determinada por una acción heurística, es decir, procesos de transformación que llevan un sentido explicativo o comprensivo. Así, la interpretación lleva la información más allá del plano semántico, de la condición potencial de “adquirir significado” al atributo de “tener sentido”. En esta fase del ciclo productivo del conocimiento, se busca identificar elementos que puedan indicar la universalidad de los fenómenos estudiados, con un enfoque en la generalización como punto central del proceso de producción de conocimiento. Como comenta Mingers^31^:

Cabe señalar que la transformación de la información en significado es intencional en un sentido fenomenológico, requiere un ser consciente. Las computadoras pueden transmitir información, pero no pueden convertirla en significado. Por otro lado, los seres humanos solo procesan significado, no información. [Traducción de: *Note that the transformation of information into meaning is intentional, in a phenomenological sense - it requires a sentient being. Computers can transmit information but cannot transform it into meaning. Conversely, human beings only process meaning, not information*].

Como hemos visto anteriormente, como resultado de una nueva ontología de la información^16^, después de superar sucesivas etapas de los límites del mundo físico-material facilitados por las TIC, podemos considerar conceptos operativos de realidades y entornos virtuales en general, y en particular, de entornos educativos y asistenciales, como pliegues del ciberespacio sobre la infosfera. Para ello, los avances de las tecnologías digitales, como la hiperconectividad, el big data, la robótica y la inteligencia artificial (IA), permiten desarrollar estrategias integradas y efectivas de observación permanente y continua producción de datos, información y conocimiento.

En resumen, una interpretación neo-pragmática crítica indica que la tecnología resulta del conocimiento, pero no depende únicamente de este para constituirse como producto de la cultura humana^19^. En este sentido, la tecnología no surge directamente de la información validada, a veces llamada “evidencia científica”, ya que necesita que se evalúen sus impactos y se establezcan sus aplicaciones con propiedad y claridad. Desde una perspectiva realista-crítica, por lo tanto, reconociendo la importancia crucial de los procesos de transformación de la información en conocimiento, la tecnología no resulta solo de la simple recopilación de datos e información en un modelo lineal de innovación, sino del conocimiento socialmente contextualizado aplicado de manera creativa^21^.

A partir de esta plataforma epistemológica, en este momento es relevante explorar los fundamentos etimológicos, históricos y teóricos de los conceptos relacionados de presencia, telepresencia, metapresencia y metapresencialidad, considerando perspectivas de aplicación en el campo de la salud.

## PRESENCIA-TELEPRESENCIA-METAPRESENCIA: METAPRESENCIALIDAD

En las lenguas neolatinas, la palabra “presencia” tiene su origen en el francés antiguo “*presence*” (siglo XII), derivado del latín “*praesentia*”, que significa la condición de “estar en un lugar y no en otro”. El término latino comprende el participio presente de “*praesse*”, que a su vez incorpora el prefijo “prae-” y la raíz “-esse”, literalmente “ser o estar antes o adelante de”^39^. El antónimo de “presencia” es la palabra “ausencia”, derivada del latín “*absentia*”. “Presente” también implica la dimensión temporal momentánea; la situación en la que una persona o algo puede “estar presente”, es decir, existir en el ahora, en este momento actual, en el tiempo presente, en una condición objetiva (algo) o subjetiva (persona). En este caso, el término “presencia” forma parte de la serie semántica “pasado-presente-futuro”, que es la base para definir la temporalidad en la cultura occidental^29^.

La cuestión de la presencia humana ha sido valorada en la investigación sobre los fundamentos, procesos e impactos de las TIC, en paralelo al desarrollo de dispositivos y soluciones tecnológicas de realidad virtual y, en estos entornos, para la realización de la telepresencia o presencia virtual^40^^,^^41^. El creciente interés en la investigación sobre la presencia virtual se confirmó temprano en el escenario tecnocientífico internacional, al punto de que en 1992 se creó en el MIT una revista dedicada a estudios sobre “sistemas directamente relacionados con la interfaz humano-máquina o con el sentido de la presencia”, que hoy se titula “*Presence: Virtual and Augmented Reality*”. En 2002, se fundó la International Society for Presence Research (ISPR) con el objetivo de fomentar “la investigación académica relacionada con el concepto de (tele)presencia”.

El concepto de “copresencia” (*copresence*) fue propuesto inicialmente por Erving Goffman^42^ para el análisis de estudios etnográficos del cuerpo y su aparato sensorial involucrado en interacciones sociales en la vida cotidiana, desde la perspectiva del interaccionismo simbólico. Este marco de referencia se retomó en el concepto de “presencia social” (*social presence*) en los primeros estudios de psicología social de las telecomunicaciones^43^, y posteriormente se utilizó en la construcción de modelos teóricos de “presencia mediada por tecnologías inmersivas”^44^.

Los conceptos de “*tele-presence*” y “*tele-operator*” se formularon por primera vez a principios de la década de 1980^45^^,^^46^. En la década de 1990, surgieron los conceptos de “*virtual presence*”^47^, “*sense of presence*”^48^ y “*depth of presence*”^49^. Posteriormente, se propusieron distinciones semánticas entre “*natural presence*”, “*sensory presence*” y “*telepresence*” como conceptos dentro de una *teoría de la presencia espacial*^50^, además de los conceptos de “*hyperpresence*”^51^^,^^52^ y “*holistic presence*”^41^. Actualmente, se observa una reafirmación de la matriz del interaccionismo simbólico en el concepto de “*enactive copresence*”^53^ y una síntesis general aplicada a la clínica en el concepto “*social telepresence*”^54^. Cabe destacar que todo este proceso de desarrollo y establecimiento conceptual se ha centrado en instituciones académicas y científicas del mundo anglosajón, como el MIT, Stanford, Harvard, Oxford y Cambridge.

El prefijo “meta-” proviene del antiguo griego μετά, que significa “más allá”, “después” o “detrás de”. En los dos primeros sentidos, corresponde al prefijo latino “trans-”^39^. En el ámbito filosófico, adquirió el significado de “trascendencia” cuando Aristóteles lo utilizó para designar la metafísica como una de las ramas de la filosofía clásica. En el discurso de las ciencias naturales, denota sustitución o alternancia, teniendo como antagonistas u opositores a los prefijos “orto-” y “para-”^55^. En el glosario de la filosofía del conocimiento y de las ciencias del lenguaje, conlleva la connotación de reflexivo o recursivo, incidente sobre sí mismo o sobre otras cosas del mismo tipo, y se refiere a un nivel superior o más allá. Por ejemplo, el metalenguaje es el lenguaje que analiza otro lenguaje, los metadatos son datos que clasifican o codifican otros datos, el metanálisis es un análisis de análisis, la metaciencia es una ciencia que estudia las ciencias y la metacognición es el conocimiento sobre otros conocimientos.

El uso del prefijo “meta-”, en referencia a los fenómenos de la presencia humana y para recalificar conceptos derivados de la presencialidad, se produjo tardíamente en el campo de las tecnociencias. La primera referencia al término compuesto “meta-presencia” se hizo en un estudio sobre presencia múltiple y compromiso en juegos digitales, que empleó una “escala de meta-presencia”. Sin embargo, esta referencia fugaz no resultó en una exploración teórica ni en una elaboración conceptual sistemática en el campo de las ciencias de la computación y de los datos, ni hubo una traducción o acercamiento a las ciencias sociales y humanas aplicadas interesadas en temas de comunicación, educación y salud.

Edmundo Balsemão-Pires^56^, en un análisis semántico del fenómeno de la individuación y del papel de la imaginación en la producción ideológica de la conciencia social, utiliza la palabra “meta-presencia” casi incidentalmente para referirse a una presencia imaginaria que sustituye la ausencia física de un sujeto convertido en símbolo en una circunstancia dada. Para este autor, la imaginación suspende el carácter objetivo de la presencia en la percepción para reemplazarlo por una forma ideal, como una “meta-presencia”.

En ese mismo año, Ricardo Cuberos^57^ propuso un modelo teórico para estudiar el impacto provocado por la introducción de la telefonía celular en los procesos simbólicos de la cognición microespacial que resultarían en deslocalizaciones de seres y sujetos. En la base de esta formulación, que anticipa la noción de metapresencialidad como un concepto, hay una triple clasificación de modos de cognición: presencial, telepresencial, metapresencial^57^. En esta propuesta, la *cognición presencial* valora la realidad localizada, incluye una conciencia cinestésica propia y se realiza mediante la observación cara a cara de otras personas, incluyendo el cuidado de objetos de propiedad personal. La *cognición telepresencial* implica la comunicación interpersonal con el interlocutor al otro lado de una línea telefónica, sin el manejo de gestos como modos cinestésicos de expresión. Finalmente, la *cognición metapresencial* es “generada a partir del manejo del hecho comunicativo mediado por el celular, como colocación del auricular en el rostro y distanciamiento interpersonal en busca de privacidad”^57^. Así, considerando las tres categorías de cognición propuestas (y delimitadas por límites variables debido a las respuestas de retroalimentación entre ellas), la evolución temporal y espacial de cada situación puede indicarse en forma de curvas correspondientes a cada patrón de presencia. Cuberos^57^ menciona “una profunda permanencia telepresencial, con una mayor distribución, cobertura espacial y distanciamiento en el recorrido del individuo y una breve migración a la metapresencialidad”.

Aun sin una referencia explícita a estas contribuciones iniciales, los términos metapresencia y metapresencialidad también se han utilizado en estudios críticos en el campo de las artes. Al explorar las imposibilidades visuales de la ciencia ficción en la película “Solaris” de Tarkovsky, Leon Marvell^58^ utiliza el término metapresencia como atributo del océano alienígena de Solaris. En un estudio sobre la influencia de la diáspora africana y lo que denomina como metacuestionamiento en la dramaturgia estadounidense, Lyndon Gill^59^ alinea la noción de “metapresencia de la negritud” con una cierta “metapresencia de la *queerness*” en la obra de James Baldwin.

Estas propuestas se encuentran en un nivel proto-conceptual, sin mayores preocupaciones por la precisión epistemológica, en un proceso creativo de formalización teórica. Una formalización más detallada de la idea de “metapresencia” y del concepto de “metapresencialidad” fue presentada por Marcus Alves^60^, con el objetivo de analizar la condición “en línea” en el contexto de los estudios de cibercultura. Aplicando directamente la teoría de los simulacros de Jean Baudrillard^61^, este autor propone que, contrario a lo que podría pensar el sentido común, la cibernética no elimina la “presencia” en el mundo social, sino que hace imposible una ausencia radical. Para Alves^60^, el concepto de “presencia” necesita ser completamente revisado, considerando que las bases experienciales de la presencia social imponen una sensación de “presencia del cuerpo biológico” como condición para la percepción de la existencia consciente en el mundo cibernético.

En la actualidad, debido a la capacidad técnica de emisión, recepción y transmisión de señales para crear imágenes mentales como si fuera una conciencia sensorial, el cuerpo físico-material ausente adquiere una forma fantasmagórica de presencia virtual, una “metapresencia”. En los procesos de comunicación mediados por tecnologías de la información, la metapresencia funciona de manera análoga a una ilusión óptica, como el ilusionismo. Esto se logra a través de un proceso técnico de simulación que Alves^60^ denomina “duplicación del yo en un soporte digital”, creando y manteniendo “una apariencia espectral del individuo que siempre está en línea, siempre en red, un simulacro de su presencia”. Y añade^60^: “La falta de evidencia de ausencia se convierte en argumento suficiente para la creación de lo que aquí llamamos metapresencia”.

En el mundo cibernético, las formas técnicas de telecomunicación determinan una cierta “deslocalización de la identidad” que, como forma política, promueve nuevas modalidades de presencia (telepresencia o metapresencia) en la condición o estado en línea, mediante la reducción o desmembramiento (a través de la codificación y transcripción digital) del cuerpo físico en el acto comunicativo^28^. Producido por la autonomía de los medios cibernéticos, el estado en línea se viabiliza como un doble, un *döppelgänger* o avatar, una forma de simulacro al cual se dirige la señal sin necesidad de certificación de validez, materialidad o incluso sincronía (facilitada por el avance en dispositivos digitales de hiper-almacenamiento de datos)^60^. Para Alves^60^, el estado en línea “es el significante de presencia lanzado con cierta exclusividad por la cibernética, como un receptáculo activo de enunciados”, que se estructura sobre una incapacidad de las personas involucradas en un proceso comunicativo mediado por tecnologías digitales para distinguir entre presencia o ausencia, únicamente en función de referencias sensoriales materiales. Como resultado de esta condición en línea y deslocalizada, las matrices mentales que antes permitían distinguir ausencia y presencia son superadas por otras referencias basadas en una metapresencia constante (que él denomina “meta-permanencia”) marcada por la virtual imposibilidad de la propia ausencia.

Estos intentos de aplicar teóricamente la idea de metapresencia en las ciencias de la comunicación y campos afines se basan ostensiblemente en un marco epistemológico-teórico del norte global (basado en el pensamiento de intelectuales célebres como Marshall McLuhan, Walter Benjamin, Michel Foucault, Jacques Derrida, Giorgio Agamben, Vlem Flusser, Jean Baudrillard) y hacen solo referencias fugaces o fragmentadas a las matrices de pensamiento contra-colonial. A pesar del esfuerzo inicial por presentar una proposición tipológica, se puede notar en estos análisis la transición semántica de una notación descriptiva (la metapresencia) a la delimitación de un atributo (la metapresencialidad). Sin embargo, ninguna de estas iniciativas explicita la intención de desarrollar y abordar el concepto de metapresencialidad en una connotación pragmática operativa, integrada a una perspectiva política.

Entre 2012 y 2017, estuve al frente del proceso de concepción e implementación de la Universidad Federal del Sur de Bahía (UFSB), una institución basada en la integración social y que invertía en una sólida base tecnológica como forma de ampliar su relevancia social^62^. En la práctica, se desarrolló y aplicó una concepción activa de metapresencia como alternativa crítica al concepto de educación a distancia (EAD), buscando crear un modelo innovador de educación abierta, inclusiva y socialmente referenciada. A través de las tecnologías digitales, se buscó superar las limitaciones de la presencia física material, mediante estrategias de reestructuración del espacio pedagógico y de la relación de enseñanza-aprendizaje a través del acceso remoto en línea sincrónico a través de la metapresencia y el acceso digital asincrónico; considerando la desconstrucción de la inconsistencia lógica en la distinción entre lo real-material y lo digital-virtual mediante una práctica proactiva, creando y probando entornos inmersivos y situaciones reales-virtuales en el proceso concreto de implementación de la nueva institución universitaria. Con este objetivo, se diseñaron entornos de enseñanza-aprendizaje como espacios y lugares colectivos, en situaciones reales, virtuales o reales-virtuales, en los cuales el estudiante podía motivarse a experimentar y explorar problemas y cuestiones reales, potenciales o preprogramados, fomentando actitudes de autoaprendizaje integradas en las demandas formativas^63^. En este experimento, la antigua noción de entornos virtuales de enseñanza fue superada por el concepto de *espacio metapresencial de aprendizaje* (EMA), concretando la idea de “muro virtual” o “ventana digital”, como interfaz inmersiva, visual y auditiva, que permite el almacenamiento y recuperación de materiales y registros pedagógicos generados en cualquier punto de la red digital de esa nueva institución universitaria.

Buscando una elaboración conceptualmente rigurosa del proyecto de la UFSB, designamos la presencia -que es al mismo tiempo real (física) y virtual (aunque mediada por tecnologías digitales, sigue siendo real)- de los sujetos en entornos virtuales de aprendizaje como “metapresencia”, tomando la “metapresencialidad” como el concepto que fundamenta esta formulación. Este esfuerzo de co-creación teórica y metodológica implica una apropiación consciente del polisémico prefijo “meta-”, con la consiguiente propuesta de los conceptos de metapresencia y metapresencialidad aplicados al diseño de un modelo de educación superior abierto, inclusivo y territorializado. Cabe destacar que el desarrollo de esta serie de conceptos en un contexto práctico, determinado por las demandas de un proceso de creación institucional, a pesar de las similitudes y convergencias, se produjo de manera totalmente independiente de las propuestas de Balsemão-Pires^56^ y Cuberos^57^, mencionadas anteriormente. Finalmente, en el proceso de viabilización de la deseada integración tecnosocial llevada a cabo en la UFSB, la noción de metapresencia en cierto sentido se concreta en el concepto operativo de metapresencialidad que, en los términos de Althusser^64^, reúne todas las condiciones para ser considerado como un “concepto en estado práctico” resultante de una “práctica teórica” fundamental para la creación institucional en curso.

## METAPRESENCIALIDAD EN LA SALUD DIGITAL

En el contexto global contemporáneo, que tiene un fuerte impacto en el ámbito local y nacional, las concepciones operativas de la “realidad” impulsadas por las tecnologías digitales son de interés para delimitar el campo de la salud digital, en los planos simultáneos de la atención médica y la formación de profesionales de la salud. En el contexto de la crisis sanitaria de la pandemia del covid-19, ha aumentado el interés en los campos de la educación y la salud por las tecnologías digitales que generan percepciones inmersivas, reemplazando la presencia física por formas sensoriales de presencia digitalmente reconstruida a través del acceso remoto.

En los espacios sociales e institucionales de la salud digital, las tecnologías digitales de salud tienen el potencial de operar tanto a nivel individual y clínico como a nivel colectivo y poblacional^65^. En el ámbito clínico, los programas informáticos se utilizan en la atención médica individual a través de la telemedicina, haciendo uso de medios de comunicación integrados y tecnologías que pueden llevar a cabo diversas tareas de apoyo al diagnóstico y tratamiento, ya sea local o a distancia. Desde la perspectiva de la salud pública, las mega bases de datos, alimentadas por redes digitales cada vez más rápidas y poderosas, conectadas en tiempo real a través de sistemas interconectados de satélites y cables de fibra óptica, y analizadas por dispositivos de inteligencia artificial, sin lugar a dudas, mejoran la efectividad en el ámbito de las políticas de salud pública. Estos procesos de apropiación sociotécnica de las tecnologías digitales de salud, sin duda, plantean cuestiones y dilemas de naturaleza filosófica, tecnocientífica, ético-política y sociocultural^66^.

Con algunos ajustes, es posible desarrollar, probar y aplicar las tecnologías digitales de salud, generadoras de inmersión, profundización y ubicuidad, que faciliten la accesibilidad y sean efectivas para la construcción de conocimientos y prácticas de manera integrada en múltiples proyectos de salud digital, como entornos de atención preventiva, curativa y rehabilitadora. Estas son necesarias para la planificación, gestión y evaluación de los procesos de atención médica. En este sentido, el concepto práctico de metapresencialidad tiene un gran potencial para ser adoptado como base para una teoría crítica de las tecnologías digitales en el ámbito de la salud. Para que esto suceda, una etapa de ajuste conceptual será seguramente estratégica en este proceso de transposición desde su origen en la educación hacia la salud digital. Primero, veamos en qué consiste el campo semántico de la salud digital.

Históricamente, varios términos pueden considerarse precursores del concepto de “salud digital”. La expresión “informática médica”, que luego fue reemplazada por “informática en salud”, enfatizaba la referencia a los equipos informáticos utilizados en el procesamiento de datos clínicos. En el ámbito de la salud pública, desde una dimensión poblacional, dos expresiones cobraron mayor visibilidad, primero “información en salud” y luego “sistemas de información en salud”, al resaltar la importancia de las bases de datos en salud. Recientemente, Moraes y Fornazin^65^ propusieron la expresión “información y tecnología de la información en salud” (y el acrónimo correspondiente, ITIS) para incorporar el conjunto de temas y prácticas relacionadas con las tecnologías digitales de salud.

La primera referencia al término “salud digital” data del año 1995, en la presentación de un programa de apoyo a la gestión hospitalaria^67^. Ya en 2001, Gunther Eysenbach^68^ definía de manera amplia la idea de salud digital, prácticamente superponiéndola a lo que se llegó a denominar *e-salud*. Según el glosario de la Estrategia Mundial de Salud Digital 2020-2025^69^, el concepto de salud digital (*dHealth*) proviene de dos nociones prácticas: e-salud (*eHealth*) y m-salud (*mHealth*). La siguiente fase de la salud digital se caracterizó por la búsqueda de movilidad completa, desde una perspectiva individual del cuidado, lo que resultó en el concepto de m-salud o “salud móvil”, en inglés: *mHealth*^70^. Con la amplia difusión de dispositivos móviles con acceso a Internet, entre otros avances tecnológicos, se hizo técnicamente posible implementar estrategias de monitoreo remoto de las condiciones de salud individuales, lo que resultó en la mejora de las estrategias de promoción de la salud a nivel colectivo.

La Organización Mundial de la Salud (OMS) considera la salud digital, en términos generales, de manera descriptiva como “el uso de tecnologías digitales de información y comunicación para la salud”, en una amplia gama de aplicaciones, desde registros médicos digitales, diagnósticos remotos y telemedicina hasta aplicaciones móviles y dispositivos portátiles individuales (*wearables*), incluyendo tecnologías, equipos, protocolos, herramientas y aplicaciones de inteligencia artificial (IA) para enfoques diagnósticos, terapéuticos, paliativos y de rehabilitación de la salud^69^. Sin embargo, la salud digital va más allá del simple uso de tecnologías digitales en el ámbito clínico o epidemiológico, acogiendo una gran cantidad de iniciativas de investigación y desarrollo tecnológico^71^. De hecho, representa una oportunidad de transformación profunda en la forma en que abordamos los desafíos del sector de la salud, involucrando una visión integrada que abarca a diferentes actores y perspectivas. Este enfoque colectivo y colaborativo es fundamental para asegurar que las tecnologías digitales se utilicen de manera efectiva y ética, beneficiando la salud de la población y promoviendo el avance del conocimiento y la práctica en el campo de la salud. En Brasil, en el contexto del Sistema Único de Salud (SUS), el concepto de salud digital se define en el documento “Estrategia de Salud Digital para Brasil 2020-2028” como redes de información y comunicación en salud, disponibles en línea para el público en general, incluidos los profesionales de la salud, con el potencial de fortalecer el trabajo en equipo, la articulación en red y la integración en todos los niveles del sistema para mejorar la atención médica local y global^72^.

Varias perspectivas y modelos teóricos se han propuesto para ayudar a orientar el desarrollo e implementación de intervenciones digitales en salud. En principio, prometen construir una base heurística capaz de comprender los factores complejos que pueden influir en la implementación de intervenciones en salud digital, incluyendo factores organizativos, adherencia, aceptación y satisfacción del usuario, cuestiones técnicas y competencias de los agentes^7^. Sin embargo, la literatura relevante que se ha presentado como referencia tecnológica de la salud digital se centra en abordajes principalmente descriptivos y en cierta medida superficiales, aunque panorámicos, de la infraestructura física (conectividad, equipos y dispositivos auxiliares), estructuras (redes, sistemas y bases de datos), herramientas (historias clínicas electrónicas, registros de autoservicio y protocolos), procesos operativos (programas, aplicaciones y rutinas) y aplicaciones de técnicas digitales para la resolución de problemas o la dirección de intervenciones en situaciones de salud. En resumen, parecen interesados solo en el mapeo superficial de usos y aplicaciones, útil para explorar espacios y mercados para el lanzamiento de nuevos productos, pero insuficiente para una comprensión más profunda y sólida de un nuevo campo de conocimientos y prácticas en formación, y mucho menos para concebir y orientar una rearticulación política de los ecosistemas de salud y sus intersecciones.

Como excepción, encontré un enfoque prometedor en el sentido de considerar el contexto actual de las tecnologías digitales de salud, pero aún descriptivo y limitado en términos de historicidad y potencial transformador. Este enfoque comprende las modelizaciones ecológicas del proceso de innovación en el campo de la salud digital^7^. En este modelo ecosistémico, se intenta incluir prácticamente todos los conceptos, valores, aplicaciones, tendencias, vectores y etiquetas relacionados con las tecnologías digitales de salud en una red complicada (pero no necesariamente compleja), con el objetivo de “mapear” el campo de la salud digital como un ecosistema institucional, una especie de matriz de redes-flujos-actores de inspiración remota en Latour.

Propongo definir la salud digital como un campo emergente de conocimiento, técnicas y prácticas, cuya influencia se extiende a través de múltiples dimensiones sociales interconectadas. Moraes y Fornazin^65^ se refieren al menos a cinco de estas dimensiones: 1) administración gubernamental; 2) atención clínica; 3) salud colectiva, destacando el uso intensivo de grandes bases de datos (*big data*) en plataformas digitales; 4) la incorporación de las tecnologías de la información y la comunicación en el complejo económico industrial de la salud; y 5) la reflexión crítica y la producción de conocimientos, tecnologías e innovación a partir de observaciones en el contexto de la salud global. El concepto de tecnologías digitales de salud se amplía al incorporar capacidades y competencias intelectuales, habilidades técnicas (reconocer y definir problemas, plantear soluciones, introducir modificaciones en el proceso de trabajo, transferir y generalizar conocimientos, pensar estratégicamente) y el ámbito subjetivo, dimensiones inmateriales determinantes del conocimiento colectivo en acción, por lo que el campo de la salud digital se confirma como multidimensional.

La salud digital valora la “competencia tecnológica crítica” para su constitución como campo de conocimientos, prácticas y técnicas^73^. La concepción de “competencia tecnológica” adoptada aquí se aleja del sentido común y busca una formulación epistemológicamente más rigurosa y ética, y políticamente más humanizada^74^. Con el objetivo de una calificación más teórico-crítica y menos instrumental del término “tecnológico”, se incorporan variantes procesuales y simbólicas de prácticas sistematizadas de aplicación del conocimiento científico^75^. Particularmente en el campo de la salud, se ha propuesto un espectro de prácticas tecnoasistenciales que va más allá del dualismo entre “tecnologías duras” y, en el polo opuesto, las llamadas “tecnologías blandas”^76^.

En el contexto de este trabajo, propongo considerar los programas y estrategias de este amplio campo, en los siguientes bloques de integración tecnosocial de la salud digital:

- Implementación de tecnologías organizativas (estructurales y de proceso) en todos los niveles, sectores y dimensiones del sistema de salud, a través de estrategias de gobernanza, recientemente revitalizadas por políticas públicas impulsadas por el Estado y basadas en conocimiento científico.
- Incorporación de tecnologías de atención, prevención y rehabilitación en diferentes niveles de la red de atención médica, en forma de protocolos, consensos y directrices terapéuticas probadas en términos de eficacia, eficiencia y efectividad.
- Adopción de tecnologías de automatización robótica para llevar a cabo intervenciones quirúrgicas de alta precisión, ya sea en el lugar o en línea, en entornos virtuales de atención médica.
- Introducción, a diferentes escalas, de tecnologías de diagnóstico en forma de pruebas automatizadas de uso masivo y sistemas de diagnóstico estructurados y remotos, especialmente aquellos relacionados con tecnologías digitales.
- Apropiación de tecnologías digitales de conectividad para llevar a cabo actividades de interacción clínica en lugares remotos o en entornos virtuales de atención médica.

En el plano de la práctica, el experimento institucional de la UFSB nos permitió superar el modelo convencional de entorno físico de aprendizaje a través de la mediación de tecnologías digitales de información y conectividad, donde la distancia geográfica dejaba de existir y el espacio remoto se convertía en metapresencial, una parte funcional del entorno expandido. En este experimento institucional, probamos una concepción práctica de metapresencialidad que buscaba incorporar todas las formas de presencia, ya sea real-material-concreta o digital-virtual-informacional, orientada hacia el compromiso y la motivación, mucho más efectiva que la educación a distancia. A pesar de la evidente y rápida aplicabilidad de este concepto para el necesario proceso de formación de individuos para una cultura de salud digital, por analogía podemos extender el concepto de espacios metapresenciales de atención para incluir espacios metapresenciales de cuidado.

En el plano semántico, surgen preguntas: Si, como hemos visto, existe toda una literatura que valida diferentes conceptos de “presencia virtual”, “co-presencia”, “tele-presencia” e incluso “hiper-presencia”, ¿qué ventajas habría en la incorporación creativa de los conceptos de metapresencia y metapresencialidad? ¿Por qué optar por el prefijo “meta-” en lugar de reforzar la idea de “tele-”? ¿Por qué multiplicar la terminología si ya hay convergencias y consensos en torno a la idea de telepresencia? En el caso de las tecnologías digitales de salud, estas preguntas serían aún más pertinentes, ya que los términos telemedicina y telesalud aparentemente ya tienen una gran aceptación en el campo de la salud digital. Sin embargo, la mera existencia de un patrón semántico establecido, en cierta medida inercial, por sí sola no justificaría la adopción de una concepción poco rigurosa y superficial, limitada a la dimensión físico-geográfica de la posición de los sujetos del aprendizaje (docentes y estudiantes) o de los sujetos de atención (usuarios y profesionales de la salud). Como vimos anteriormente, la polisemia del término metapresencia y su derivado metapresencialidad abre un horizonte de significados como “receptáculo activo de enunciaciones” que incluye la condición en línea, reterritorializada, local-presencia virtualizada, mucho más allá de lo denotado por el prefijo “tele-”, que simplemente significa lejos, remoto, a distancia.

Más allá de los planos prácticos y semánticos, se pueden identificar algunas diferencias de forma y posibles ventajas epistemológicas o heurísticas en el uso del concepto de “metapresencialidad” en comparación con los campos de la informática, la educación y la salud. Propongo hacer esto en referencia (o contraste) a temas representativos de otros modelos de apropiación sociotécnica de las TIC, como la idea de “metaverso” en la realidad virtual y las prácticas de “telesalud” en la salud digital.

Recientemente, el prefijo “meta-” ha ganado notoriedad en el escenario global al ser adoptado por una de las principales empresas de tecnología actual, que en una operación de cambio de marca pasó a llamarse “Meta” y denominó a su principal programa de negocios como “metaverso”^28^. La idea de “metapresencia”, como hemos visto, se integra críticamente en la propuesta del “metaverso”, sin abandonar sus referencias simultáneas a la interfaz real-virtual, local-remoto (tele-) y la condición en línea, tal como lo analizó Alves^60^. Alejándose de esta recuperación oportunista de la idea de “metaverso”, que consagra el individualismo y el aislamiento a través de las gafas de la realidad virtual, el concepto de “metapresencialidad” se beneficia de la polisemia del prefijo “meta-” para enfatizar una propuesta integradora de las interfaces real-virtual, digital-material e individual-colectiva, orientada hacia la solidaridad y el compartir de los espacios metapresenciales de atención médica, especialmente en relación con las prácticas que componen lo que se conoce como “telesalud”.

La telesalud implica una densificación tecnológica, una apropiación sociotécnica y una integración tecnosocial de las tecnologías digitales de la información y la comunicación en forma de plataformas en línea a través de teleconsultas (atención médica a distancia), telecirugías (intervenciones quirúrgicas robóticas) y teleconsultoría (consultas con especialistas), lo que sin duda contribuye al avance y consolidación de una nueva generación de modelos de atención médica, reconfigurando el campo de la salud^77^. Además, la introducción de sistemas de telemedicina en línea permitirá a los médicos enviar recetas electrónicas directamente a la red de farmacias, programar citas a través de aplicaciones y automatizar los itinerarios terapéuticos, lo que reducirá significativamente la burocracia y el problema de las largas filas de pacientes en las instituciones de salud. Este modelo de atención emergente, aún en proceso de concepción e implementación en diferentes partes del mundo, ha impulsado (y se ha beneficiado de) innovaciones en las tecnologías digitales de la información y la comunicación y avances en los conceptos de salud digital, especialmente ampliando su dimensión participativa, ya que los propios usuarios pueden generar información, acceder a registros de atención médica y utilizar sistemas autónomos de comunicación entre pacientes y profesionales de la salud, fácilmente accesibles para evaluación diagnóstica y pronóstica, así como para el monitoreo de pacientes^78^.

## COMENTARIOS FINALES

La inminente introducción de las tecnologías digitales en salud en los sistemas de salud públicos, como el Sistema Único de Salud (SUS) en Brasil, sin duda implicará un profundo proceso de transformación digital. La concepción de “sistemas locales de salud” (SILOS), difundida por la Organización Panamericana de la Salud (OPS) durante las últimas décadas del siglo XX, principalmente en América Latina^79^, define el lugar como una ubicación espacial territorializada, la distancia como una separación geográfica y la presencia como la coexistencia de seres materiales (humanos y no humanos) en el mismo entorno físico y en el mismo momento temporal. Subyacente a esta concepción convencional y anacrónica, se encuentra la suposición de que el proceso de atención médica es el resultado de una relación intersubjetiva directa, guiada por la relación clínica y que en última instancia se individualiza^74^^,^^75^. Desde entonces, estos conceptos se han considerado requisitos previos para una atención médica pública resolutiva y eficiente.

Sin embargo, desde una perspectiva crítica, conceptos de este tipo expresan condiciones restrictivas o limitantes de ese complejo proceso material, social y psicológico de enculturación que, en el campo de la educación, se ha denominado proceso de enseñanza-aprendizaje y que, en el ámbito de la salud, se ha denominado relación médico-paciente o, en su versión más común, relación usuario-servicio de salud. En cualquiera de los casos, se refieren al encuentro interpersonal entre el individuo que sufre (por enfermedad o ignorancia) y los profesionales capacitados para llevar a cabo prácticas, aplicar técnicas y movilizar tecnologías que faciliten el proceso educativo o la atención médica. El uso de diferentes formas de metapresencia podría superar los desafíos impuestos por una concepción restrictiva de la realidad material y de la presencia física como requisitos indispensables para la atención médica, lo que aumentaría los costos, reduciría la escala y limitaría el acceso, factores que promueven desigualdades e inequidades en la salud.

Para una comprensión crítica de la problemática de la salud digital y sus instituciones, estrategias y dispositivos vuelvo a recurrir a Milton Santos^12^ quien, al postular una geografía potencialmente descolonizadora, plantea una profunda interrogación de las concepciones de lugar como ubicación espacial, distancia como mera separación geográfica y presencia como coexistencia de seres materiales en un mismo entorno físico y en el mismo momento temporal. A partir de este marco teórico, podemos cuestionar estas nociones como determinantes de los procesos interactivos en la educación y el cuidado de las personas. Esta tríada (distancia-presencia-lugar) se basa en una concepción lineal y exclusivamente física del tiempo y el espacio en la vida social cotidiana, en los entornos educativos y en los ecosistemas de salud. Desde un enfoque que se estableció como normalizado, estas nociones a menudo se han considerado requisitos previos para una educación eficaz y, por analogía, para una atención médica humanizada y resolutiva. En esta perspectiva convencional, basada en una concepción lineal de la temporalidad, la noción de sincronicidad puede ser tomada como una categoría explicativa de la presencia material de los actores en el proceso de atención, según una tipología que los clasifica, en una dicotomía temporal, como acciones sincrónicas o asincrónicas^80^. Esta concepción, de origen pedagógico, se apoya en la noción de sincronicidad como descriptor de la presencia física simultánea (problemática y con logística compleja, socialmente discriminada) de todos los actores (y actantes) relevantes en el proceso de atención médica. Subyacente a esta concepción se encuentra la suposición de que los actos de atención médica son simplemente el resultado de una relación interpersonal singular basada en la transmisión interpersonal de contenidos y valores que, en última instancia, se individualiza.

Como conclusión provisoria de esta reflexión en curso, es importante mencionar la cuestión del sujeto significativo para el campo de la salud digital. Como he señalado en diferentes ocasiones^81^^,^^82^^,^^83^, en el mundo contemporáneo estamos experimentando un proceso de creación no solo de nuevos mundos y nuevos campos de conocimiento, prácticas y técnicas, sino también de nuevos seres/sujetos. En los niveles macro y microsociales de esta nueva socialidad, se trata de perfiles de sujetos recreados por la traducción digital, seres humanos, transhumanos y poshumanos^84^. Esta formulación se inspira en la noción de cíborg propuesta hace décadas por Donna Haraway^85^, para quien la sociedad tecnológica promueve la emergencia de una nueva especie de persona, con dos o más esferas de vida en paralelo, viviendo una existencia real y concreta, con contacto físico entre seres y productos materiales, en interacción con una existencia real y virtual mediada por tecnologías digitales. Esta concepción se ha actualizado con la idea de “cuerpo electrónico” propuesta por Stefano Rodotà^86^ para indicar nuevos sujetos de derechos relacionados con realidades ampliadas, cuyas vidas virtuales están compuestas por redes sociales, correos electrónicos, blogs, canales de vídeo, en interacción con otras personas y bienes virtuales en el mundo imaginario (pero no menos real) que inicialmente se llamó ciberespacio y luego se denominó metaverso.

El concepto de metapresencialidad sin duda puede contribuir a la delimitación epistemológica del nuevo campo de la salud digital, como interfaz y componente de la salud colectiva, que es simultáneamente un campo académico-disciplinario, político y de acción tecnológica. De esta manera, espero que este esfuerzo conceptual ayude en la concepción e implementación de políticas públicas orientadas hacia la calidad y equidad en la atención médica en Brasil y América Latina.
