# Supplementary material for: Metapresencialidad: concepto fundante de una teoría crítica de la salud digital
Source: Salud Colect. 2023 Oct 19;19:e4655. [Article in Spanish] doi: 10.18294/sc.2023.4655 (PMC11930329; doi:10.18294/sc.2023.4655)
Supplement: Translation

# Translation: Metapresentiality: a foundational concept for a critical theory of digital health

**Authors:** Naomar de Almeida Filho

**Affiliations:** 1 PhD en Epidemiología. Profesor titular jubilado, Instituto de Saúde Coletiva, Universidade Federal da Bahia. Profesor visitante, Instituto de Estudos Avançados, Universidade de São Paulo. Consultor Senior, Secretaria de Informação e Saúde Digital, Ministério da Saúde, Brazil. naomaralmeida@gmail.com

**Keywords:** Information Theory, Digital Technology, Digital Health, Telemedicine

## Abstract

In this text, I propose the concept of “metapresentiality” as a fundamental element for a Critical Theory of Digital Health. First, I present the concepts of technique, technology, and technical object, central to the theories of Álvaro Vieira Pinto and Milton Santos. Secondly, based on Luciano Floridi’s philosophy of information, I question the relevance of the dichotomy real-material-concrete vs. digital-virtual-informational as an ontological foundation for concepts of reality, place, and presence, highlighting the notions of virtual reality and extended reality. Thirdly, I introduce an etymological-historical critique of the series presence-telepresence-metapresence, focusing on the emerging notion of meta-presentiality in the form of proto-concept and its eventual formalization as a conceptual foundation for a socio-technical appropriation and technosocial integration of digital technologies. Finally, I discuss Digital Health as a field of knowledge, techniques and practices and evaluate epistemological and pragmatic advantages of metapresentiality as a concept in the fields of computing, education and health.

## INTRODUCTION

The society that has emerged from the globalization of productive systems since the second half of the 20th century inaugurates a new historical phase of humanity. Production relations in contemporary social formations have been marked by the intensive and constant use of technologies, especially digital technologies, in all areas of human knowledge and social action.^1^ The complexity of the current world determines new forms of intervention in everyday life, efficient, agile, and flexible, carried out through diverse, modular, and ever-changing sociotechnical strategies. The emergence of these interventions in immaterial spaces and non-presential situations has occurred through systems, equipment, processes, and programs with complex functionality, classified as *information and communication technologies* (ICT), with massive and widespread implementation and an increasingly frequent use in all sectors of the global economy. Enabled by robotic automation systems, programmed in machine language, and controlled by algorithms (recently, by artificial intelligence), ICT have led to the consideration of contemporary capitalism as a “digital economy”.^2^

At the societal level, with the global diffusion of ICT in all aspects of social life, there is an increasingly proliferation of a variety of processes, products, and patterns of social use of digital technologies for the production and utilization of data, information, and knowledge. In this scenario, integrated communication devices and interpersonal connection systems (social networks, chats, blogs, etc.) foster technosocial integration processes mediated by human-machine interfaces, creating forms of sociability. As a result, human social organization and relational processes in everyday life are becoming increasingly dependent on databases, sources of information, digital networks, and electronic devices, justifying the label of the “knowledge society”^3^ that is in vogue.

The mass availability of digital technical objects and their widespread use has been considered a factor in structural unemployment, educational setbacks, social exclusion, cultural alienation, and a potential vector for mental health harm.^4^ Nevertheless, optimists argue that, by taking advantage of gaps and opportunities, political processes of democratic rationality and interactive education, when carried out competently, could mitigate risks and offset the detrimental effects of technocentrism.^5^ In this way, there is hope that ICT, with their multiple potentials, can contribute to the complete civic education of a new epistemic subject, encouraged to learn throughout life, in a supportive relationship with human communities that have become virtual and within a sustainable environment.^6^

Worldwide, the sociotechnical appropriation of knowledge and intervention experiences based on ICT has fostered the implementation of innovative health ecosystems that, rather than being mere increments, supplements, or accessories to existing forms, models, strategies, and methods, potentially represent a revolution in modes of healthcare.^7^ With the COVID-19 pandemic, there has been a significant increase in interest in digital technologies that create immersive perceptions, replacing material presence with sensory forms of remote presence, particularly in the fields of education and healthcare. Since then, *digital health technologies* (DHT) have spread in situations and contexts that mobilize large contingents of technical operators and a vast volume of information, alongside the proliferation of relevant technical objects. The delineation of this set of technical objects, techniques, technological innovations, and their operators, organized and active within spaces, institutional networks, and communities of practice, has become a new social field that can be termed *digital health.*

To address this set of issues rigorously and consistently, I propose exploring foundations and systematizing necessary, useful, and viable concepts for the construction of a critical theory of digital health, from a perspective of epistemic disobedience.^8^ In this article, I highlight one of these concepts: metapresence, as a focal point for reflection capable of shaping strategies and qualifying opportunities for the application of DHT. To some extent, albeit preliminary and limited in scope, this text represents an effort to expand, organize, and detail a brief personal communication included in the book *O futuro começa agora: Da pandemia à utopia* by Boaventura de Sousa Santos.^9^

In this process of shared theoretical construction, first, I propose to introduce the notions of technology, technique, and technical object, based on the theoretical contributions of Álvaro Vieira Pinto^10^^,^^11^ and Milton Santos.^12^^,^^13^ Secondly, in dialogue with the philosophy of information, as proposed by Luciano Floridi,^14^^,^^15^^,^^16^^,^^17^ and building upon a theory of the mode of production of technoscientific knowledge,^18^^,^^19^^,^^20^^,^^21^ I question the relevance of the dichotomy between the real-material-concrete and the digital-virtual-informational as an epistemological foundation for defining the concepts of reality, place, presence, and presentiality, emphasizing the notions of virtual reality and extended reality. Thirdly, I introduce a perspective of etymological and historical critique of the semantic series presence-telepresence-metapresence, focusing on the emerging notion of metapresentiality as a proto-concept and its potential formalization as a conceptual foundation for the sociotechnical appropriation and technosocial integration of DHT. Finally, in comparison with the proposals of the metaverse (in the field of computing) and conventional notions of distance education (in the field of education) and telemedicine (in the field of healthcare), I assess the potential epistemological, heuristic, and operational advantages of the concept of metapresentiality for the establishment of digital health as a field of knowledge, techniques, and practices.

## TECHNOLOGY, TECHNIQUE, TECHNOCENTRISM

Álvaro Vieira Pinto (1909-1987) was a polymath (physician, mathematician, physicist, demographer, translator, philosopher, social thinker, and educator) who led a generation of intellectuals representing left-leaning national-developmental thought in the late 20th century. In his extensive body of work, Vieira Pinto^10^^,^^11^^)^ proposed a philosophical, historical, and political analysis of the relationships between labor and production, nature and technique, science and culture, all tied to the process of dependent development. His critical reflections on phenomena related to the incorporation and social appropriation of techniques and technologies have been revisited and studied, particularly following the posthumous publication of *O conceito de tecnologia.*^11^ In this work, an ambitious and complex treatise on the technological era and its developments, he begins by deconstructing the expression “technological era,” which was widely circulated at that time. He uses a direct and compelling argument to criticize and refute it, stating that precisely because humans are human, they have always lived in technological eras. As humans produce increasingly complex and sophisticated technologies, they become more dependent on them, in a dialectical and tendentially conflictive relationship. Nowadays, technology plays an indispensable role in social functioning and labor relations, often simplifying the challenges of technological progress as purely “technical” aspects.^11^

In the contemporary imaginary, as an ideological construct, the term “technology” constitutes a metonymy (more precisely a synecdoche), used to refer to diverse things and topics: material technical objects (tools and electronic equipment), operated by techniques (fixed or self-programmable programs and protocols), and, as a condition for the viability of these objects and processes, digital information and connectivity technologies. To overcome this technological ideology, Vieira Pinto^11^ identifies the need for greater precision in the conceptualization of what “technology” is, distinguishing it from the concepts of technique, instrument, and product (which were later conceptualized as “technical objects”). For him, the term “technique” refers to the way in which human productive acts are carried out, materializing in instruments, machines, and artifacts that transform nature, humanizing it through culture.

On the other hand, the term “technology” unfolds into two concepts and three common uses, with some degree of semantic overlap. Firstly, in a theoretically etymologically precise reference, the concept of technology signifies the science of technique (*technê + logos*) or knowledge about technique. Secondly, in common social discourse, in which Vieira Pinto^11^ highlights a somewhat naive view of “technology,” the notion of technology often gets reduced to technique or sets of techniques, equating process and discourse. Thirdly, as a derivation of this lay connotation, the anthropological conception of technology refers to all systematic knowledge produced and accumulated historically by humankind throughout its existence, encompassing the set of techniques developed and appropriated in a specific historical period.^10^ Fourth, the dominant conception of the nature of technology, representative of uncritical and anachronistic thinking, refers to the ideology of technique.^11^ The concept of technique as an ideology allows for a critical understanding of technocentrism, defined by Seymour Papert^22^ as the overvaluation of technology, placing it at the center of human activity and giving it the role of the “principal solver” of humanity’s problems. This latter notion of technique relates to the social imaginary of the contemporary world, capable of turning technology into mythology, as indicated by Vieira Pinto.^11^

Technocentrism presents itself to the alienated human subject, as it fails to recognize that:

...the machine is nothing more than its work, the product of its inner purposes, [...] and believes, on the contrary, that it must allow itself to be possessed by technology because only in this way can it acquire a human name and essence, that of a technician.^11^

To deconstruct this ideological trap of alienating technocentrism, in a passionate and militant tone, Vieira Pinto^11^ encourages us to:

...break the infernal circle of a false totality in which the dominators want to confine us, under the pretext that we all participate in the same world, unified by science and technology, which have now reached such a degree of progress that no one can reject them, but neither has the right to give free rein to creation on their own.

In order to critically approach the concepts, practices, strategies, and devices of digital technologies, we can also turn to the theory of technique in the social realm by the geographer, epistemologist, and critical thinker Milton Santos [1926-2001]. In his effort to recreate the epistemology of human and social sciences as a whole, Milton Santos^12^^,^^13^ proposes an approach that considers space as an inseparable set of systems of objects and systems of actions. Space is a mixture, a hybrid, a complex, a geographical environment composed of different forms and contents materialized in multiple totalities. What has always existed from these totalities is a geographical environment that transforms historically, which for two or three centuries was referred to as the “technical or machinic environment” and which today we can designate as the “technical-scientific-informational environment.”

For Milton Santos,^13^ the “main form of relationship between human beings and nature, or rather, between human beings and their environment, occurs through technique.” As the basis for this assertion, techniques are understood as a set of instrumental and social means through which humans carry out their lives, simultaneously producing and creating space. In Milton’s theory, the relationship between space and the technical phenomenon encompasses all manifestations of technique, including the techniques of action themselves, especially those that produce technical objects. It involves approaching the technical phenomenon as a complex totality, avoiding being dazzled by techniques defined in abstract. It’s not just about considering “so-called production techniques, or as others prefer, industrial techniques, i.e., specific techniques viewed as a means to achieve a specific result”.^13^

Milton Santos^13^ emphasizes the importance of distinguishing between specific techniques - when examined in their singularity - and technique as a total phenomenon. Consequently, one cannot conceive of a rigid separation between “a geographical environment on one hand, and a technical environment on the other”.^13^ Thus, in the “technical-scientific-informational environment,” techniques should be viewed not only in their material aspect but also in their immaterial aspects, through their own history as systems that mark epochs. Santos continues:

To discuss the present and the current conditions for the realization and transformation of space, I assume from the outset the knowledge of what constitutes the current technical system and how, based on the conditions of the current technique, an informational technique, the material and political conditions were established that authorized the production of a planetary intelligence.

In the contemporary context, digital information technologies are fundamental for recreating and demarcating new geographical and geopolitical landscapes. In this regard, he comments that “information today plays a role analogous to the role played in the past by energy” as it becomes the tool to connect different parts of an abstract territory that, thanks to information coverage, has become less local and more global, allowing for the “presence of absent bodies”.^13^ Leveraging the advances in mobility and connectivity provided by information technology, which define the contemporary world, the dominant classes, paradoxically, are participating less and less in the local world of territories and, therefore, “see little of the city and of the world”.^12^

In this complex and ever-changing context, technique plays a central role, and technology is one of the main systems of actions present in the territories of the world globalized by the cultural hegemony of capitalist West. In this current stage of capitalism, where ICT act as organizing mechanisms for the manufacturing, distribution, and marketing of goods, products, and services, the adjective “digital” (from the Latin *digitus*, meaning finger, as counting with fingers was the primary primitive method used for counting things) has been used in the numerical sense to designate systems and processes produced from the encoding of signals, data, and information, as well as any effects they produce.^11^ Therefore, the term “digital technology” refers to techniques (procedures, protocols, guidelines) and technical objects (equipment, devices) whose functionality and effective operation depend on programs and languages (operating systems, programming languages, algorithms) enabled by logical systems or sequences of commands formulated in binary codes.

With the development of methods and devices for digitization, compression, and integration of signals, images, and sounds, the adjective “virtual” has been used to name technologies that create artificial or simulated environments through immersive means. The rapid advancement of these technologies, especially in the entertainment (games) and training (simulators) sectors, has expanded the capacity for processing, compression, transmission, and integration of signals, leading to increasingly efficient sensory devices. These technologies disseminate solutions that produce simulation effects and modeling of environments and objects, related to sensory simulations known as “virtual reality.” Among other functions and possibilities, these objects, situations, and states allow for the redefinition of the very notion of virtuality as a property of spaces, objects, systems, and processes modeled through digital codes and syntax. Based on an assessment of these technological contexts, I propose exploring the hypothesis of the non-relevance or invalidity of the almost intuitive dichotomy, omnipresent in common sense, that contrasts the terms real-material *versus* digital-virtual, set in absolute and exclusive terms. From a philosophical perspective, this is a fundamental ontological question that I will analyze in the next section.

## REALISMS, REALITIES, VIRTUALITIES

To substantiate this analysis, I initially sought to undertake a critical review of the work of the Italian philosopher Luciano Floridi,^14^^,^^15^^,^^16^^,^^17^ who formulated a philosophy of information based on what could be called “ontological informational realism”. This proposal opens and organizes an entire field of research on categories and concepts that, in the contemporary world, focus on the determinants, processes, and impacts of cybernetics and computer technologies, with a focus on data sciences and information science.

In his own words,^15^ it is a:

...philosophical field that encompasses the critical investigation of the conceptual nature and principles of information, including its dynamics, uses, and sciences, and the development and application of theoretical-informational and computational methodologies to address philosophical problems.

The repertoire of research problems listed by Floridi^14^^,^^15^ encompasses five groups of topics that would be characteristic of a realist philosophy of information: information, semantics, cognition, ethics, and ontology.

When addressing the fundamental ontological question of “what is information?” Floridi^15^ points out the impossibility and ultimately the lack of relevance of a unified theory of information. In this process, he identifies three conceptions of information with a clear ontological bias: a) information as reality, as in cybernetics; b) information about reality, which would be the case of semantic information; c) reality as information, of which the genome would be an example. As the central axis of his philosophical project, Floridi prioritizes the conceptions of information as reality or about reality, which are developed in six approaches:

- Mathematical theory of data/signals: defines information in terms of numerical formalization;
- Probabilistic approach: defines information in terms of the stochastic space;
- Topological approach: defines information in terms of modal space;
- Systemic modeling: defines information in terms of processes and flows;
- Inferential approach: defines information based on the space of inferences;
- Semantic approach: emerges as the main objective of his philosophical research, enabled as an analytical-synthetic object, by defining information in terms of the space of data.

To formulate a semantic theory of information, Floridi^14^ suggests delimiting the concepts of the ontology of information based on their respective attributes: information must be quantifiable, plausible, accumulative, storable, and transmissible. The semantic nature of information is not exclusively or necessarily linguistic, and there is an independence of semantic information from the physical medium, format, and language. In summary, verified or validated data, structured enough to be encoded in numerical bases and, most importantly of all, meaningful data, are what make semantic information concrete.^14^ The raw material of information is data, transformed according to a syntax, following the rules of a system, code, or language available to the operating subject. Data, in turn, emerge from “networks of observables”,^15^ anchored in ontological frameworks, whose verification or validation implies epistemic commitments that allow the construction of information networks and conceptual matrices.

The realm of human cognition, data appears as a condition for the production of information because by shaping knowledge capable of guiding techniques and supporting practices, information becomes a constitutive condition of knowledge and its pragmatic validity. Here arises the question of how meaningful data transformed into information attain the value of truth, which opens the door to a theory of truth based on the semantics of information as the raw material of knowledge. In this dimension, Floridi^17^ proposes a “map of semantic information” as a transformative sequence that defines what is called “intelligence.” Consequently, the question arises as to whether cognition can be understood in terms of information processing or if an interpretive transformation is needed to move from information to knowledge.

In the ethical-value dimension, for Floridi,^15^ information technologies have the capacity to impact social systems and everyday life forms. In light of the philosophy of information, a computer ethics is required to address normative demands that arise from the dynamics of information, which often produce unintended and sometimes harmful effects. This can occur in two ways: on one hand, by identifying emerging problems in order to prevent them or at least raise awareness among professionals, politicians, and the public; on the other hand, by applying corrective measures to address the negative effects of information dynamics and social problems produced by new technologies. In this regard, Floridi^15^ mentions microethics built around some moral value of information: first, considering it as a useful resource; second, considering it as a product used to generate more information; third, in a historical perspective of the information context intertwined with cultural, social, and political contexts.

Finally, on the ontological dimension, especially regarding the relationship between data and nature and between information and reality (the natural world), Floridi’s information semantics^14^^,^^15^ aims to evaluate the quality of this relationship. To do this, it explores the possibility that the very nature of the natural world has configured ecosystems of information as an *infosphere*, a space of simultaneous material and virtual, natural and informational realities and temporalities. Hence, it is justifiable to speak of real-time in reference to the simultaneity or synchrony of digital interaction, in many ways. Floridi^15^^,^^16^ develops the idea of levels of abstraction to propose modeling as a possible link between the real and processes of semanticization, which are ultimately processes of constructing reality. As we saw earlier, to become information, data needs to acquire meaning, so modeling information produces semantic effects in constructed realities.^17^ In this way, as analyzed by González,^23^ Floridi’s thesis of informational realism encompasses the modeling process as a dialectical interface between data (supporting the processes of semanticizing the real) and information (linking semanticization to pragmatic processes capable of generating knowledge).

With the declared intention of surpassing epistemologies of representation and interpretation inherited from Cartesian rationalism and its derivations, Floridi^24^ adopts a very distinctive constructivist perspective. By positing that human life is tied to relevant events in the world of language, he assumes that this connection authorizes the symbolic (the core of human language) to separate reality from the real. Capurro^25^ questions Floridi’s thesis that, constituted as a semantic operator, the human being would be capable of promoting a division between thing and symbol through linguistic mediation. This postulation had already been evaluated (and criticized) as metaphysical and neoplatonic. In any form of Platonism, whether classical or contemporary, the real is defined as what was established as a limit, and realities are constructed to try to account for the real that, in always a restricted and partial way, presents itself for semanticization. In contrast, the Aristotelian naturalistic approach, inherited by the dominant empiricism in the natural sciences, defines the real as what is and what exists, denying any valid reference to the ideal world of pure forms or paradigms. In various ways, Floridi’s structural realism of information maintains that knowledge is constructed from information, assuming that the validity of information depends on modeling processes based on data, which, in turn, originate from real observables. This issue repositions the problem of beings and connections in the physical universe; it is about verifying whether processes considered as natural, such as causality or temporal presence, indeed encompass information dynamics in an empirical reality. For Floridi,^14^ this issue breaks down into crucial questions, with the main one being the ontological difference between material reality and virtual realities.

Given the complexity of these topics and with the aim of at least organizing ways of understanding realities according to Floridi that can guide techniques and underpin practices based on a virtuality established through digital means, we can consider the following common glossary.^26^

- *Restricted Reality*: This refers to an environment in which the process of attention is limited to a direct and synchronous therapeutic relationship with the physical presence of individuals throughout the attention process, without technological mediation, information, or connectivity. Examples of this concept of restricted reality include the technoscientific and micropolitical social environment of laboratories, observatories, and research fields, as well as the care environment of hospitals, specialized clinics, outpatient services, and practice settings, which require the physical presence of actors in crucial stages of the process and do not make use of digital devices.
- *Projected Reality*: This refers to the reproduction of care or teaching-learning environments in remote locations, where the pedagogical or demonstration relationship can occur residually or asynchronously, facilitated by the use of ICT devices. This involves technological mediation for the editing, assembly, storage, and transmission of preprogrammed content. Examples of this modality include prerecorded video teleclasses or debates that are edited, illustrated, and projected in time and space, presenting an image, process, clinical situation, or pedagogical context.
- *Augmented or Extended Reality*: This refers to the extension of the physical environment in which production, communication, education, healthcare, and other forms of relational sociability take place, facilitated by the use of digital connectivity devices.^26^ In the field of healthcare, it is defined as an extension of the real and concrete care environment where therapeutic relationships are carried out directly and synchronously, and can occur through virtual presence (or telepresence), made possible by the use of digital telecommunication devices for the transmission of context, images, and sound. A simplified example of this type of environment would be the real-time transmission of a surgical procedure, a lecture, or a performance on screens and sound systems in adjacent rooms or simultaneously in remote locations, which can be even more effective in educational terms if interactive participation devices are included.
- *Virtual Reality*: This is a simulated environment that is detached from a concrete material matrix and is entirely digital. Its microecological references are transformed into signals through digital codes that, when decoded and converted back into sensory stimuli, allow for an experiential or motivational immersive relationship.^26^ Examples of VR devices can include role-playing games, video games, and programmed simulations among avatars. These can become simulations of anatomical, physiological, cellular, and molecular microenvironments, especially effective for technical and professional training in the healthcare field.

This is a topic related to the creation of immaterial realities.^27^ Both augmented reality and virtual reality involve a delocalized territoriality, enabling financial, pedagogical, or therapeutic relationships in contexts of sensory or motivational immersion, programmed by devices and production systems that condense and integrate context, image, sound, and data.^28^ As a result (or alternatively), the processes of semantic information production (in Floridi’s terminology) make it possible to produce knowledge, and in parallel cycles, the production of technologies, especially digital technologies capable of providing virtuality and its realities.

In practice, with the advent of digital sensory immersion technologies through the integration of audiovisual information, alternative models of reality once again demonstrate the lack of relevance or futility of the distinction between the real and the virtual, pointing to their overcoming through complementary, hybrid, or transgressive forms of this originally conjunctive-identitarian perspective.^29^ In this direction, Mingers and Standing^30^ note that current theories of information still have a long way to go and list a series of fundamental questions that need to be answered:

What is the ontological status of information - what exactly is it - a thing, a concept, a relation, a meaning? Is it objective, existing independently of observers or receivers, or is it subjective, created in the mind of observers on receipt of a message? […] Can there be “environmental information”, that is signs within the environment that carry information without the involvement of humans? Does information have to be true to be information (a veridical version) as Dretske and Floridi maintain? […] Does an information theory distinguish clearly between the related concepts of data, information, knowledge and meaning?

The contextualized view identifies significant potential in the ontological informational realism to address epistemological challenges and practical situations in the current techno-scientific scenario. In order to be accepted within the Eurocentric establishment, Floridi’s informational constructivism approach may conceal its distant roots in a theory of the mode of knowledge production, as subtly pointed out by Mingers.^31^ Dialectical-critical realism encompasses a theory of knowledge and scientific practice derived from dialectical materialism and methodological pragmatism (in the line of Peirce-James-Dewey-Rorty), as systematically formulated by the Indo-British philosopher and logician Ram Roy Bhaskar (1944-2014) in the late 1970s.^18^ Other significant authors in this global movement for critical realism include the American logician Donald Mertz,^32^the Australian philosopher Alan Chalmers,^33^ and the Argentine epistemologist Juan Samaja.^20^^,^^21^

Although Floridi does not acknowledge any theoretical connection to critical realism in any of its versions, his work implicitly contains a theory of modes of information-knowledge production with notable similarities to the critical epistemology of Juan Samaja (1941-2007). For Samaja,^20^^,^^21^ the attributes of events or phenomena are not really the crucial elements for constructing the object-model, the theoretical framework that enables the production of scientific knowledge, but rather the methodological and analytical praxis of the sciences, guided by the limits and barriers (or conditioning factors) of concrete reality. From Samaja’s perspective,^21^ by analogy with the theory of the mode of economic production developed by historical materialism, the techno-scientific mode of production encompasses a productive process of concepts, models, goods, and values defined by specific properties that differentiate it from the production of goods and products in general.

From the perspectives of both Bhaskar^19^ and Samaja,^20^ scientific practice involves a fundamental dialectic between systematic knowledge consolidated as theory, through organized and articulated concepts in explanatory matrices or models, and the problems generated by the ongoing reference to the practical-empirical field, i.e., in a close and inevitable interaction with the concrete and real. The construction of consistent theories based on philosophically sound and contextually relevant concepts is crucial for driving the production of knowledge in various areas of study and deriving technologies capable of advancing different spheres of application. According to Samaja,^21^^,^^34^ the development of scientific and technological knowledge occurs through a production chain that involves the transformation of data into concepts, composed of stages of transformation of the scientific object and its intermediate products, as well as its results in the form of a technological object. This crucial stage is mediated by specific indicators and methodologies that vary depending on the type of data used. In this way, concepts act as heuristic tools for understanding the phenomena under study, allowing for the generalization of explanation and the application of findings in different contexts.^21^

Following the Bhaskar-Samaja framework, I move away from the numerous conceptions of “information” prevalent in the epistemologies of the global North, particularly the empirical notion of information as content carried by signals or signs in the mathematical theory of information.^35^ In line with Høstaker,^27^ I reject the idea of the materiality of information in the concrete world, which forms the basis of theories inspired by the semantic turn of neopragmatism that have dominated the field of “information sciences”.^36^^,^^37^ On the other hand, I see promise in approaches based on critical realism that allow for a deeper understanding of socio-historical phenomena and enable the integration of information from different areas, the identification of patterns and trends, and the establishment of connections between concepts and theories as a peculiar mode of production that is structured in cycles of data to information and knowledge transformation, and then from knowledge to technique and praxis.^34^

The transition from data to information is determined by processes of analytical transformation, which involve complex organization, indexing, classification, condensation, and interpretation of data. The goal of these processes is to identify similarities in dimensions, attributes, predicates, and properties among cases in order to turn them into “information.” For data to become relevant and meaningful, it is necessary to compare them, searching for patterns and relationships that generate meaning. Through this process, data is transformed into “semantic information,” representing a higher level in the knowledge production process. As mentioned by Mingers,^31^ this can be expressed in the formula: information = data + meaning, in a model of knowledge production cycles. One of the earliest models of this kind was the Data-Information-Knowledge-Wisdom (DIKW) model developed by Ackoff.^38^ From this perspective, information is produced from data that are processed appropriately and consistently, with the goal of solving problems, answering questions, or testing hypotheses.^34^

For information to become knowledge, the information derived from data can be interpreted, related to existing theories, compared with other data, and contextualized within a broader conceptual framework. The transition from information to knowledge is determined by a heuristic action, i.e., transformation processes that carry an explanatory or comprehensive sense. Thus, interpretation takes information beyond the semantic plane, from the potential condition of “acquiring meaning” to the attribute of “making sense.” In this phase of the knowledge production cycle, the goal is to identify elements that may indicate the universality of the studied phenomena, with a focus on generalization as the central point of the knowledge production process. As commented by Mingers.^31^

Note that the transformation of information into meaning is intentional, in a phenomenological sense - it requires a sentient being. Computers can transmit information but cannot transform it into meaning. Conversely, human beings only process meaning, not information.

As we have seen above, as a result of a new ontology of information,^16^ after overcoming successive stages of the limits of the physical-material world facilitated by ICT, we can consider operational concepts of realities and virtual environments in general, and in particular, educational and healthcare environments, as folds in cyberspace over the infosphere. To achieve this, advances in digital technologies, such as hyperconnectivity, big data, robotics, and artificial intelligence (AI), enable the development of integrated and effective strategies for ongoing observation and continuous production of data, information, and knowledge.

From this epistemological platform, it is now relevant to explore the etymological, historical, and theoretical foundations of related concepts like presence, telepresence, metapresence, and metapresentiality, for considering their perspectives and applications in the field of healthcare.

## PRESENCE-TELEPRESENCE-METAPRESENCE: METAPRESENTIALITY

In Neo-Latin languages, the word “presence” originates from Old French “*présance*” (12th century), derived from the Latin *“praesentia*”, which means the condition of “being in one place and not in another”. The Latin term comprises the present participle of “*praesse*”, which, in turn, incorporates the prefix “prae-” and the root “-esse,” literally meaning “to be or exist before or ahead of”.^39^ The antonym of “presence” is the word “absence,” derived from the Latin “*absentia*”. “Presence” also implies the temporal dimension of the momentary; the situation in which a person or something can be “present”, that is, exist in the now, in this current moment, in the present time, in an objective (something) or subjective (person) condition. In this case, the term “presence” is part of the semantic series “past-present-future,” which forms the basis for defining temporality in Western culture.^29^

The question of human presence has been valued in research on the foundations, processes, and impacts of ICT, parallel to the development of virtual reality devices and technological solutions for telepresence or virtual presence.^40^^,^^41^ The growing interest in research on virtual presence was confirmed early on in the international technoscientific scenario, to the extent that in 1992, a journal dedicated to studies on “systems directly related to the human-machine interface or the sense of presence” was created at MIT, which is now titled “Presence: Virtual and Augmented Reality.” In 2002, the International Society for Presence Research (ISPR) was founded with the aim of promoting “academic research related to the concept of (tele)presence”.

The concept of “copresence” was initially proposed by Erving Goffman for the analysis of ethnographic studies of the body and its sensory apparatus involved in social interactions in everyday life, from the perspective of symbolic interactionism.^42^ This framework was later adopted in the concept of “social presence” in early studies of social psychology of telecommunications^43^, and it was subsequently used in the construction of theoretical models of “presence mediated by immersive technologies”.^44^

The concepts of “tele-presence” and “tele-operator” were first formulated in the early 1980s.^45^^,^^46^ In the 1990s, concepts like “virtual presence”,^47^ “sense of presence”,^48^ and “depth of presence”^49^ emerged. Subsequently, semantic distinctions were proposed between “natural presence”, “sensory presence” and “telepresence” as concepts within a theory of spatial presence,^50^ in addition to concepts like “hyperpresence”^51^^,^^52^ and “holistic presence”.^41^ Currently, there is a reaffirmation of the symbolic interactionism framework in the concept of “enactive copresence”^53^ and a general synthesis applied to clinical contexts in the concept of “social telepresence”.^54^ Noteworthy is that this entire process of conceptual development and establishment has been primarily centered in academic and scientific institutions in the Anglo-Saxon world, such as MIT, Stanford, Harvard, Oxford, and Cambridge.

The prefix “meta-” comes from ancient Greek μετά, which means “beyond”, “after”, or “behind.” In the first two senses, it corresponds to the Latin prefix “trans-”.^39^ In the philosophical realm, it acquired the meaning of “transcendence” when Aristotle used it to designate metaphysics as one of the branches of classical philosophy. In the discourse of the natural sciences, it denotes substitution or alternation, with the antagonists or opposites being the prefixes “ortho-” and “para-”.^55^ In the glossary of epistemology and the philosophy of language, it carries the connotation of being reflexive or recursive, acting upon itself or upon other things of the same kind, and refers to a higher or beyond level. For example, metalanguage is language that analyzes another language, metadata is data that classifies or encodes other data, meta-analysis is an analysis of analyses, metascience is a science that studies the sciences, and metacognition is knowledge about other forms of knowledge.

The use of the prefix “meta-” in reference to phenomena of human presence and to requalify concepts derived from presence occurred relatively late in the field of technosciences. The first reference to the compound term “meta-presence” was made in a study on multiple presence and engagement in digital games, which used a “meta-presence scale”. However, this fleeting reference did not result in a theoretical exploration or systematic conceptual elaboration in the field of computer and data sciences, nor was there a translation or approach to the applied social and human sciences interested in communication, education, and health-related topics.

Edmundo Balsemão-Pires,^56^ in a semantic analysis of the phenomenon of individuation and the role of imagination in the ideological production of social consciousness, uses the word “meta-presence” almost incidentally to refer to an imaginary presence that replaces the physical absence of a subject turned into a symbol in a given circumstance. For this author, imagination suspends the objective character of presence in perception and replaces it with an ideal form, like a “meta-presence”.

In that same year, Ricardo Cuberos^57^ proposed a theoretical model to study the impact caused by the introduction of mobile telephony on the symbolic processes of microspatial cognition that would result in the delocalization of beings and subjects. At the basis of this formulation, which anticipates the notion of metapresence as a concept, is a triple classification of modes of cognition: presential, telepresential, metapresential.^57^ In this proposal, *presential cognition* values localized reality, includes one’s own kinesthetic awareness, and is carried out through face-to-face observation of other people, including the handling of personal property objects. *Telepresential cognition* involves interpersonal communication with the interlocutor on the other end of a telephone line, without the use of gestures as kinesthetic modes of expression. Finally, *metapresential cognition* is “generated from the handling of the communicative fact mediated by the cell phone, such as placing the handset on the face and interpersonal distancing in search of privacy”.^57^ Thus, considering the three proposed categories of cognition (and delimited by variable boundaries due to feedback responses between them), the temporal and spatial evolution of each situation can be indicated in the form of curves corresponding to each pattern of presence. Cuberos^57^ mentions “a deep telepresential permanence, with greater distribution, spatial coverage, and distancing in the individual’s journey and a brief migration to metapresentiality”.

Even without explicit reference to these early contributions, the terms metapresence and metapresentiality have also been used in critical studies in the field of the arts. When exploring the visual impossibilities of science fiction in Tarkovsky’s film “Solaris”, Leon Marvell^58^ uses the term metapresence as an attribute of Solaris’ alien ocean. In a study on the influence of the African diaspora and what he terms as meta-questioning in American dramaturgy, Lyndon Gill^59^ aligns the notion of the “metapresence of blackness” with a certain “metapresence of queerness” in James Baldwin’s work.

These proposals are at a proto-conceptual level, with no major concerns for epistemological precision, in a creative process of theoretical formalization. A more detailed formalization of the idea of “metapresence” and the concept of “metapresentiality” was presented by Marcus Alves,^60^ aiming to analyze the “online” condition in the context of cyberculture studies. Applying Jean Baudrillard’s theory of simulacra directly,^61^ this author proposes that, contrary to what common sense might suggest, cybernetics does not eliminate “presence” in the social world but makes a radical absence impossible. For Alves,^60^ the concept of “presence” needs to be completely revisited, considering that the experiential foundations of social presence impose a sense of “presence of the biological body” as a condition for perceiving conscious existence in the cybernetic world.

Currently, due to the technical capacity to emit, receive, and transmit signals to create mental images as if it were sensory awareness, the absent physical-material body acquires a ghostly form of virtual presence, a “metapresence”. In information technology-mediated communication processes, metapresence functions analogously to an optical illusion, like illusionism. This is achieved through a technical process of simulation that Alves^60^ calls “duplicating the self on a digital support”, creating and maintaining “a spectral appearance of the individual who is always online, always networked, a simulacrum of their presence”. He adds:^60^ “The lack of evidence of absence becomes sufficient argument for the creation of what we call metapresence”.

In the cyber world, technical forms of telecommunication determine a certain “delocalization of identity” that, as a political form, promotes new modalities of presence (telepresence or metapresence) in the online condition or state, through the reduction or dismemberment (via digital encoding and transcription) of the physical body in the communicative act.^28^ Produced by the autonomy of cyber media, the online state is made possible as a double, a *döppelgänger* or avatar, a simulacrum to which the signal is directed without the need for certification of validity, materiality, or even synchrony (facilitated by advancements in digital data storage devices).^60^

For Alves,^60^ the online state “is the signifier of presence launched with certain exclusivity by cybernetics, as an active receptacle of utterances”, which is structured upon the incapacity of individuals involved in a communicative process mediated by digital technologies to distinguish between presence or absence solely based on material sensory references. As a result of this online and delocalized condition, the mental matrices that once allowed the distinction between absence and presence are surpassed by other references based on a constant metapresence (which he calls “meta-permanence”) marked by the virtual impossibility of one’s own absence.

These attempts to theoretically apply the idea of metapresence in the fields of communication sciences and related areas are overtly based on a Northern Global epistemological-theoretical framework (influenced by renowned intellectuals like Marshall McLuhan, Walter Benjamin, Michel Foucault, Jacques Derrida, Giorgio Agamben, Vlem Flusser, Jean Baudrillard) and make only fleeting or fragmented references to counter-colonial thought matrices. Despite the initial effort to present a typological proposition, there can be observed in these analyses the semantic transition from a descriptive notation (metapresence) to the delineation of an attribute (metapresentiality). However, none of these initiatives explicitly state the intention to develop and address the concept of metapresentiality in an operational pragmatic connotation, integrated into a political perspective.

Between 2012 and 2017, I led the conception and implementation of the Universidade Federal do Sul da Bahia (UFSB), an institution based on social integration and heavily invested in a strong technological foundation as a means to foster its social relevance.^62^ In practice, an active conception of metapresence was developed and applied as a critical alternative to the concept of distance education, aiming to create an innovative model of open, inclusive, and socially-referenced education. Through digital technologies, we sought to overcome the limitations of physical material presence by restructuring the pedagogical space and the teaching-learning relationship through synchronous online remote access via metapresence and asynchronous digital access. This involved deconstructing the logical inconsistency in distinguishing between the real-material and the digital-virtual through proactive practice, creating and testing immersive environments and real-virtual situations in the concrete implementation of the new university institution. With this goal in mind, we designed teaching-learning environments as collective spaces and places, in real, virtual, or real-virtual situations, where students could be motivated to experiment and explore real, potential, or pre-programmed problems and issues, fostering self-learning attitudes integrated into the educational demands.^63^ In this experiment, the old notion of virtual teaching environments was surpassed by the concept of a *metapresential learning space*, embodying the idea of a “virtual wall” or “digital window” as an immersive, visual, and auditory interface that allows for the storage and retrieval of pedagogical materials and records generated anywhere in the digital network of this new university institution.

In seeking a conceptually rigorous elaboration of the UFSB project, we designated the presence - which is both real (physical) and virtual (although mediated by digital technologies, it remains real) - of individuals in virtual learning environments as “metapresence”, with “metapresentiality” as the concept that underpins this formulation. This effort of theoretical and methodological co-creation involves a conscious appropriation of the polysemic prefix “meta-”, leading to the proposal of the concepts of metapresence and metapresentiality applied to the design of an open, inclusive, and territorially-based higher education model. It’s worth noting that the development of this series of concepts in a practical context, driven by the demands of an institutional creation process, despite similarities and convergences, occurred entirely independently of the proposals of Balsemão-Pires^56^ and Cuberos^57^ mentioned earlier. Finally, in the process of realizing the desired technosocial integration carried out at UFSB, the notion of metapresence in a sense crystallizes in the operational concept of metapresentiality, which, in the terms of Althusser,^64^ gathers all the conditions to be considered as a “concept in practical state” resulting from a fundamental “theoretical practice” for the ongoing institutional creation.

## METAPRESENCE IN DIGITAL HEALTH

In the contemporary global context, which has a significant impact on local and national levels, the operational conceptions of “reality” driven by digital technologies are of interest in delineating the field of digital health, on both the simultaneous fronts of medical care and the training of healthcare professionals. In the context of the ongoing COVID-19 pandemic, there has been a growing interest in the fields of education and health in digital technologies that generate immersive perceptions, replacing physical presence with digitally reconstructed forms of sensory presence through remote access.

In the social and institutional spaces of digital health, digital health technologies have the potential to operate at both the individual and clinical levels, as well as at the collective and population levels.^65^ In the clinical realm, software programs are used in individual healthcare through telemedicine, making use of integrated communication media and technologies that can perform various tasks to support diagnosis and treatment, whether locally or remotely. From a public health perspective, massive databases, fueled by increasingly fast and powerful digital networks, connected in real-time through interlinked satellite and fiber optic cable systems, and analyzed by artificial intelligence devices, undeniably enhance the effectiveness of public health policies. These processes of socio-technical appropriation of digital health technologies undoubtedly raise philosophical, technoscientific, ethical-political, and sociocultural questions and dilemmas.^66^

With some adjustments, it is possible to develop, test, and apply immersive, deepening, and ubiquitous digital health technologies that facilitate accessibility and are effective for integrated knowledge and practice construction across multiple digital health projects, including preventive, curative, and rehabilitative care environments. These technologies are necessary for the planning, management, and evaluation of healthcare processes. In this regard, the practical concept of metapresentiality has great potential to be adopted as the foundation for a critical theory of digital technologies in the field of healthcare. To make this happen, a conceptual adjustment phase will likely be strategic in the process of transposing it from its origin in education to digital health. But first, let’s explore the semantic field of digital health.

Historically, several terms can be considered precursors to the concept of “digital health”. The expression “medical informatics,” later replaced by “health informatics,” emphasized the reference to computer equipment used in clinical data processing. In the field of public health, from a population dimension, two expressions gained greater visibility, first “health information” and then “health information systems”, highlighting the importance of health databases. Recently, Moraes and Fornazin^65^ proposed the term “information and information technology in health” (and the corresponding acronym, ITIS) to incorporate the set of topics and practices related to digital health technologies.

The first reference to the term “digital health” dates back to 1995, in the presentation of a hospital management support program.^67^ As early as 2001, Gunther Eysenbach^68^ broadly defined the concept of digital health, virtually overlapping it with what came to be called e-health. According to the glossary of the Global Digital Health Strategy 2020-2025,^69^ the concept of digital health (dHealth) derives from two practical notions: eHealth and mHealth. The next phase of digital health was characterized by the pursuit of complete mobility, from an individual care perspective, resulting in the concept of mhealth or “mobile health”.^70^ With the widespread availability of mobile devices with internet access, among other technological advancements, it became technically feasible to implement strategies for remote monitoring of individual health conditions, leading to improved public health promotion strategies.

The World Health Organization (WHO) broadly describes digital health as “the use of digital information and communication technologies for health” in a wide range of applications, from digital medical records, remote diagnostics, and telemedicine to mobile apps and individual wearable devices, including technologies, equipment, protocols, tools, and artificial intelligence (AI) applications for diagnostic, therapeutic, palliative, and rehabilitation approaches to health.^69^ However, digital health goes beyond the mere use of digital technologies in clinical or epidemiological settings, encompassing a multitude of research and technological development initiatives.^71^ In fact, it represents an opportunity for profound transformation in how we address the challenges of the healthcare sector, involving an integrated vision that encompasses different actors and perspectives. This collective and collaborative approach is essential to ensure that digital technologies are used effectively and ethically, benefiting the health of the population and promoting advances in knowledge and practice in the field of health. In Brazil, in the context of the *Sistema Único de Saúde* (SUS) the concept of digital health is defined in the document “Digital Health Strategy for Brazil 2020-2028” as information and communication networks in health, available online to the general public, including healthcare professionals, with the potential to strengthen teamwork, network coordination, and integration at all levels of the system to improve local and global healthcare.^72^

Several perspectives and theoretical models have been proposed to help guide the development and implementation of digital health interventions. In principle, they promise to build a heuristic foundation capable of understanding the complex factors that can influence the implementation of digital health interventions, including organizational factors, user adherence, acceptance, and satisfaction, technical issues, and agent competencies.^7^ However, the relevant literature presented as a technological reference for digital health primarily focuses on mainly descriptive and somewhat superficial, albeit panoramic, approaches to physical infrastructure (connectivity, equipment, and auxiliary devices), structures (networks, systems, and databases), tools (electronic health records, self-service records, and protocols), operational processes (programs, applications, and routines), and the application of digital techniques to problem-solving or intervention management in health situations. In summary, it seems interested only in the superficial mapping of uses and applications, useful for exploring spaces and markets for the launch of new products, but insufficient for a deeper and more solid understanding of a new field of knowledge and practice in formation, even less for conceiving and guiding a political rearticulation of health ecosystems and their intersections.

As an exception, I found a promising approach that considers the current context of digital health technologies, but it remains descriptive and limited in terms of historicity and transformative potential. This approach involves ecological modeling of the innovation process in the field of digital health.^7^ In this ecosystemic model, an attempt is made to include virtually all concepts, values, applications, trends, vectors, and labels related to digital health technologies in a complicated (but not necessarily complex) network, with the aim of “mapping” the field of digital health as an institutional ecosystem, a kind of matrix of networks-flows-actors remotely inspired by Latour.

I propose defining digital health as an emerging field of knowledge, techniques, and practices whose influence extends across multiple interconnected social dimensions. Moraes and Fornazin^65^ refer to at least five of these dimensions: 1) government administration; 2) clinical care; 3) public health, with a focus on the intensive use of big data in digital platforms; 4) the integration of information and communication technologies into the health industry complex; and 5) critical reflection and the production of knowledge, technologies, and innovation based on observations in the context of global health. The concept of digital health technologies expands to incorporate intellectual capabilities and competencies, technical skills (recognizing and defining problems, proposing solutions, making modifications to work processes, transferring and generalizing knowledge, thinking strategically), and the subjective realm, immaterial dimensions that determine collective knowledge in action, making the field of digital health multidimensional.

Digital health values “critical technological competence” for its constitution as a field of knowledge, practices, and techniques.^73^ The conception of “technological competence” adopted here deviates from common sense and seeks a more epistemologically rigorous, ethical, and humanized formulation, as well as a more theoretically critical and less instrumental qualification of the term “technological,” incorporating processual and symbolic variants of systematic practices of applying scientific knowledge^75^. Particularly in the field of health, a spectrum of techno-assistive practices has been proposed that goes beyond the dualism between “hard technologies” and, at the opposite pole, so-called “soft technologies”.^76^

In the context of this work, I propose considering the programs and strategies in the following blocks of techno-social integration in digital health:

- Implementation of organizational technologies (structural and process) at all levels, sectors, and dimensions of the healthcare system through governance strategies, recently revitalized by state-driven public policies based on scientific knowledge.
- Incorporation of care, prevention, and rehabilitation technologies at different levels of the healthcare network in the form of protocols, consensus, and therapeutic guidelines tested for efficacy, efficiency, and effectiveness.
- Adoption of robotic automation technologies to perform high-precision surgical interventions, either on-site or online, in virtual healthcare environments.
- Introduction, at different scales, of diagnostic technologies in the form of mass-use automated tests and structured remote diagnostic systems, especially those related to digital technologies.
- Appropriation of digital connectivity technologies to carry out clinical interaction activities in remote locations or in virtual healthcare environments.

In the dimension of practice, the institutional experiment at UFSB allowed us to surpass the conventional model of physical learning environments through the mediation of digital information and connectivity technologies, where geographical distance ceased to exist, and remote space became metapresential, a functional part of the expanded environment. In this institutional experiment, we tested a practical conception of metapresentiality that aimed to incorporate all forms of presence, whether real-material-concrete or digital-virtual-informational, oriented towards engagement and motivation, much more effective than distance education. Despite the evident and rapid applicability of this concept to the necessary process of educating individuals for a digital health culture, by analogy, we can extend the concept of metapresential care spaces to include metapresential care spaces.

On the semantic dimension, questions arise: If, as we have seen, there is a literature that validates various concepts of “virtual presence,” “co-presence,” “tele-presence,” and even “hyper-presence,” what advantages would there be in the creative incorporation of the concepts of metapresence and metapresentiality? Why choose the prefix “meta-” instead of reinforcing the idea of “tele-”? Why multiply terminology if there are already convergences and consensus around the idea of telepresence? In the case of digital health technologies, these questions would be even more pertinent, as terms like telemedicine and telehealth seemingly already have broad acceptance in the field of digital health. However, the mere existence of an established semantic pattern, to some extent inertial, alone would not justify the adoption of an unrigorous and superficial conception, limited to the physical-geographical dimension of the position of learning subjects (teachers and students) or care subjects (healthcare users and professionals). As we have seen before, the polysemy of the term metapresence and its derivative metapresentiality opens up a horizon of meanings as an “active receptacle of enunciations” that includes the online condition, reterritorialized, local-virtualized presence, far beyond what is denoted by the prefix “tele-”, which simply means distant or remote.

Beyond the practical and semantic levels, some differences in form and potential epistemological or heuristic advantages can be identified in the use of the concept of “metapresence” in comparison to the fields of informatics, education, and health. I propose to do this in reference (or contrast) to representative topics from other sociotechnical appropriation models of ICT, such as the idea of the “metaverse” in virtual reality and the practices of “telehealth” in digital health.

Recently, the prefix “meta-” has gained notoriety on the global stage as it was adopted by one of the leading technology companies today, which rebranded itself as “Meta” and named its main business program as the “metaverse”.^28^ The idea of “metapresence,” as we have seen, critically integrates into the proposal of the “metaverse,” without abandoning its simultaneous references to the real-virtual interface, local-remote (tele-), and the online condition, as analyzed by Alves.^60^ Moving away from this opportunistic recovery of the “metaverse” idea, which consecrates individualism and isolation through virtual reality goggles, the concept of “metapresentiality” benefits from the polysemy of the prefix “meta-” to emphasize an integrative proposal of real-virtual, digital-material, and individual-collective interfaces, oriented towards solidarity and sharing in metapresential spaces of healthcare, especially in relation to the practices that make up what is known as “telehealth.”

Telehealth involves technological densification, sociotechnical appropriation, and technosocial integration of digital information and communication technologies in the form of online platforms through teleconsultations (remote medical care), telesurgeries (robotic surgical interventions), and teleconsulting (consultations with specialists), undoubtedly contributing to the advancement and consolidation of a new generation of healthcare models, reshaping the field of healthcare.^77^ Additionally, the introduction of online telemedicine systems will enable doctors to send electronic prescriptions directly to the pharmacy network, schedule appointments through applications, and automate therapeutic itineraries, significantly reducing bureaucracy and the issue of long patient queues at healthcare institutions. This emerging care model, still in the process of conception and implementation in various parts of the world, has driven (and benefited from) innovations in digital information and communication technologies and advances in the concepts of digital health, especially expanding its participatory dimension, as users themselves can generate information, access medical records, and use autonomous communication systems between patients and healthcare professionals, easily accessible for diagnostic and prognostic evaluation, as well as patient monitoring.^78^

## FINAL COMMENTS

The imminent introduction of digital health technologies into public healthcare systems, such as the *Sistema Único de Saúde* (SUS) in Brazil, will undoubtedly entail a profound process of digital transformation. The concept of “local health systems” (LHS), disseminated by the Pan American Health Organization (PAHO) during the last decades of the 20th century, primarily in Latin America,^79^ defines place as a territorialized spatial location, distance as geographical separation, and presence as the coexistence of material beings (human and non-human) in the same physical environment and at the same moment in time. Underlying this conventional and anachronistic conception is the assumption that the healthcare process results from a direct intersubjective relationship guided by the clinical relationship and ultimately individualized.^74^^,^^75^ Since then, these concepts have been considered prerequisites for effective and efficient public healthcare.

However, from a critical perspective, concepts like these express restrictive or limiting conditions of that complex material, social, and psychological process of enculturation, which in the field of education has been termed the teaching-learning process, and in the realm of health, has been referred to as the doctor-patient relationship or, in its most common version, the user-health service relationship. In either case, they refer to the interpersonal encounter between the individual suffering (from illness or ignorance) and the professionals qualified to carry out practices, apply techniques, and mobilize technologies that facilitate the educational process or medical care. The use of various forms of metapresence could overcome the challenges imposed by a restrictive conception of material reality and physical presence as indispensable requirements for medical care, thereby increasing costs, reducing scale, and limiting access, factors that promote health inequalities and inequities.

For a critical understanding of the issue of digital health and its institutions, strategies, and devices, I once again turn to Milton Santos,^12^ who, by proposing a potentially decolonizing geography, raises a profound questioning of the concepts of place as spatial location, distance as mere geographical separation, and presence as the coexistence of material beings. From this theoretical framework, we can question these notions as determinants of interactive processes in education and the care of individuals. This triad (distance-presence-place) is based on a linear and exclusively physical conception of time and space in everyday social life, educational environments, and health ecosystems. From a perspective that was established as standardized, these notions have often been considered prerequisites for effective education and, by analogy, for humane and resolute healthcare. In this conventional perspective, based on a linear conception of temporality, the notion of synchronicity can be taken as an explanatory category for the material presence of actors in the care process, classified in a temporal dichotomy as synchronous or asynchronous actions.^80^ This conception, rooted in pedagogy, relies on the notion of synchronicity as a descriptor of simultaneous physical presence (problematic and logistically complex, socially discriminated) of all relevant actors (and actants) in the healthcare process. Underlying this conception is the assumption that medical care actions are simply the result of a singular interpersonal relationship based on the interpersonal transmission of content and values that ultimately become individualized.

As a provisional conclusion to this ongoing reflection, it is important to mention the question of the significant subject within the field of digital health. As I have noted on several occasions,^81^^,^^82^^,^^83^ in the contemporary world, we are experiencing a process of not only creating new worlds and new fields of knowledge, practices, and techniques but also of new beings/subjects. At both the macro and microsocial levels of this new sociality, we encounter profiles of subjects recreated by digital translation, including humans, transhumans, and posthumans.^84^ This formulation is inspired by the notion of the cyborg proposed decades ago by Donna Haraway,^85^ in which technological society promotes the emergence of a new kind of person with two or more spheres of life running in parallel. They live a real and concrete existence with physical contact among beings and material products while interacting with a real and virtual existence mediated by digital technologies. This conception has been updated with the idea of the “electronic body” proposed by Stefano Rodotà^86^ to indicate new subjects with rights related to expanded realities. Their virtual lives are composed of social networks, emails, blogs, video channels, interacting with other people and virtual assets in the imaginary (but no less real) world that was initially called cyberspace and later referred to as the metaverse.

The concept of metapresence undoubtedly can contribute to the epistemological delineation of the new field of digital health as an interface and component of collective health. This field is simultaneously an academic-disciplinary, political, and technological action field. In this way, I hope that this conceptual effort will assist in the conception and implementation of public policies aimed at quality and equity in healthcare in Brazil and Latin America.
